# Supplementary material for: Patient preferences in genetic newborn screening for rare diseases: study protocol
Source: BMJ Open. 2024 Apr 19;14(4):e081835. doi: 10.1136/bmjopen-2023-081835 (PMC11056621; doi:10.1136/bmjopen-2023-081835)
Supplement: Author's manuscript [file bmjopen-2023-081835.draft_revisions.pdf]

BMJ Open

BMJ Open is committed to open peer review. As part of this commitment we make the peer review history of every article we publish publicly available.

When an article is published we post the peer reviewers' comments and the authors' responses online. We also post the versions of the paper that were used during peer review. These are the versions that the peer review comments apply to.

The versions of the paper that follow are the versions that were submitted during the peer review process. They are not the versions of record or the final published versions. They should not be cited or distributed as the published version of this manuscript.

BMJ Open is an open access journal and the full, final, typeset and author-corrected version of record of the manuscript is available on our site with no access controls, subscription charges or pay-per-view fees (http://bmjopen.bmj.com).

If you have any questions on BMJ Open's open peer review process please email info.bmjopen@bmj.com

BMJ Open

Patient Preferences in Genetic Newborn Testing for Rare diseases: study protocol

Journal:	*BMJ Open*
Manuscript ID	bmjopen-2023-081835
Article Type:	Protocol
Date Submitted by the Author:	08-Nov-2023
Complete List of Authors:	MARTIN, Sylvia; Uppsala Universitet, Center for Research and Bioethics<br>Angolini, Emanuele; Ospedale Pediatrico Bambino Gesù, Research Unit of Neuromuscular and Neurodegenerative Disease<br>Audi, Jennifer; Takeda Pharmaceuticals International AG<br>Bertini, Dr. Enrico; Ospedale Pediatrico Bambino Gesù, Research Unit of Neuromuscular and Neurodegenerative Disease<br>Bruno, Lucia Pia; University of Siena, Medical Genetics; Telethon Institute of Genetics and Medicine<br>Coulter, Joshua; Pfizer Global Pharmaceuticals<br>Ferlini, Alessandra; University of Ferrara, Department of Medical Sciences<br>Fortunato, Fernanda; University of Ferrara, Department of Medical Sciences<br>Frankova, Vera; Charles University First Faculty of Medicine<br>Garnier, Nicolas; Pfizer Inc<br>Grauman, Åsa; Uppsala Universitet, Center for Research and Bioethics<br>Gross, Edith; EURORDIS<br>Hauber, Brett; Pfizer Inc<br>Hansson, Mats; Uppsala Universitet, Center for Research and Bioethics<br>Kirschner, Janbernd ; University of Freiburg<br>Knieling, Ferdinand; Erlangen University Hospital<br>Kyosovksa, Gergana; Bulgarian Association for Personalised Medicine<br>Ottombrino, Silvia; Ospedale Pediatrico Bambino Gesù, Research Unit of Neuromuscular and Neurodegenerative Disease<br>Novelli, Antonio; Ospedale Pediatrico Bambino Gesù, Research Unit of Neuromuscular and Neurodegenerative Disease<br>Raming, Roman; Erlangen University Hospital<br>Sansen, Stefaan; Sanofi-Aventis SA, Department of Medical Biotechnologies<br>Saier, Christina; Faculty of Medicine, Department of Neuropediatrics and Muscle Disorders<br>Veldwijk, Jorien; Erasmus Universiteit Rotterdam
Keywords:	GENETICS, QUALITATIVE RESEARCH, Patient-Centered Care, Patient Participation, Patient Reported Outcome Measures

Patient Preferences in Genetic Newborn Testing for Rare diseases: study protocol

Authors: Sylvia Martin[1*], Angolini Emanuele[14], Jennifer Audi[5], Enrico Silvio Bertini[14], Lucia Pia Bruno[7,8], Joshua Coulter[3], Alessandra Ferlini[12], Fernanda Fortunato[12], Vera Frankova[9], Nicolas Garnier[3], Åsa Grauman[1], Edith Gross[13], , Brett Hauber[3], Mats Hansson[1.], Janbernd Kirschner[10], Ferdinand Knieling[6], Gergana Kyosovksa[4], Silvia Ottombrino[14], Antonio Novelli[14], Roman Raming[6], Stefaan Sansen[8], Christina Saier[10], Jorien Veldwijk [1,2].

For peer review only

Word count: 5205 words

1. Center for Research and Bioethics (CRB), Uppsala University, Sweden, Husargatan 3, BMC, entrance A11.

2. Erasmus School of Health Policy & Management, Erasmus University Rotterdam, P.O.Box 1738, 3000 DR Rotterdam, The Netherlands.

3. Pfizer, 235 E 42nd St, New York, USA.

4. Bulgarian Association for Personalised Medicine, 45 Bacho Kiro str., 1202 Sofia, Bulgaria.

5. Takeda Pharmaceuticals International AG, Zurich, Switzerland

6. Departmenf of Pediatrics and Adolescent Medicine, Universitätsklinikum Erlangen, Friedrich-Alexander-Universität (FAU) Erlangen-Nürnberg, Erlangen, Germany.

7. Medical Genetics, University of Siena, Siena, Italy

8. Med Biotech Hub and Competence Center, Department of Medical Biotechnologies, University of Siena, Siena, Italy.

9. Institute for Medical Humanities, Charles University First Faculty of Medicine, Karlovo náměstí 40, 128 00 Prague 2, Czech Republic

10. Department of Neuropediatrics and Muscle Disorders, Medical Center – University of Freiburg, Faculty of Medicine, Freiburg, Germany

11. Sanofi, Diegem, Belgium.

For peer review only - http://bmjopen.bmj.com/site/about/guidelines.xhtml

12. Unit of Medical Genetics, Department of Medical Sciences, University of Ferrara, Via Fossato di Mortara, 74, 40122 Ferrara (Italy)

13. EURORDIS, Plateforme Maladies Rares,96, rue Didot, 75014 Paris (France).

14. Research Unit of Neuromuscular and Neurodegenerative Disease, Ospedale Pediatrico Bambino Gesu, Roma, Lazio (Italy).

* Sylvia Martin is the corresponding author: Center for Research and Bioethics (CRB),Uppsala University, Sweden, Husargatan 3, BMC, entrance A11.

sylvia.martin@crb.uu.se

For peer review only

For peer review only - http://bmjopen.bmj.com/site/about/guidelines.xhtml

Abstract:

Introduction: Rare diseases collectively impact over 30 million people in Europe, most individual conditions have a low prevalence which resulted in a lack of research and expertise in this field, especially regarding genetic new-born screening (gNBS). As there is increasing recognition of the importance of incorporating patients' needs and general public perspectives into the shared-decision making process regarding gNBS, the current protocol describes a study measuring preferences of the expecting parents. This study is part of the Innovative Medicine Initiative project Screen 4 Care which aims at shortening the diagnostic journey for rare diseases by accelerating diagnosis for patient living with rare diseases through genetic newborn screening (gNBS) and digital technologies such as Artificial Intelligence (AI) and Machine Learning (ML **Methods and analysis:** A mixed method approach will be conducted to assess preferences of (1) expecting parents seeking genetic testing (2) expecting parents from general population in two countries (Germany and Italy). Focus groups and interviews using the nominal group technique and ranking exercises will be performed (qualitative phase). Their results will inform the treatment of attributes to be assessed via a survey and a discrete choice experiment (DCE). The total recruitment sample will be 2084 participants (approximatively 1000 participants in each country for the survey part). A combination of thematic qualitative and logit-based quantitative approaches will be used to analyze the preferences. **Ethics and dissemination**: This study has been approved by the Erlangen University Ethics Committee (22-246_1-B), the Freiburg University Ethics committee (23-1005-S1-AV) and for clinical centers in Italy (University of FerraraCE: 357/2023/Oss/AOUFe, and Hospedale Bambino Gesu: No.2997 of 2 November 2023, Prot. No. _902), and approved for data storage and handling at Uppsala University (2022-05806-01).

Keywords: genetic newborn screening, rare disease, patient preference, discrete choice experiment.

For peer review only

Strengths and Limitations

- This study has been developed by an international expert team together with industry and patient representatives to provide an example of a rigorously designed 2 stepped preferences study.

-This study will inform both clinical research and patient advocates stakeholders contributing to gNBS for rare diseases field.

-This study will collect data from a significantly large sample size across several populations, allowing comparison of gNBS uptake preferences, psychological variables and socio-demographic information in two European countries.

- Hypothetical choices may be different that actual choices (participants willing to answer the survey might not be representative of the general population).

-Study elements might be complex (cross cultural elements, potential issues in translations, recruitment populations differences) and prone to misunderstanding even if high involvement of patient's representatives in the development of the study material will reduce these risks.

-This study will recruit participants from different countries where the in-place gNBS procedure are differently implemented in the general care settings.

For peer review only

For peer review only - http://bmjopen.bmj.com/site/about/guidelines.xhtml

Introduction

There are more than 7,000 known rare diseases (RD), conditions that affect one, or less than one, individual in 2,000 (point prevalence of <1/1 000 000 in more than 84% of RD [1]). Patients living with rare a disease typically face an arduous journey to proper diagnosis, enduring on average eight years of countless physician consultations [2]. Time to diagnosis is a stressful period during which patients are faced with inconclusive test outcomes, possible misdiagnoses, and thereby ineffective treatments and healthcare resource utilisation [3].

Although rare diseases collectively impact over 30 million people in Europe, most individual conditions have a low prevalence [4]. This has resulted in a lack of research and expertise in this field. Difficulties in the process of receiving an accurate diagnosis and uncertainty around experienced symptoms continue to place a heavy burden on people living with rare diseases, their families, caregivers, physicians, and society as a whole [5,6]. Currently, there is increasing recognition of the importance of incorporating patients' needs and perspectives into the shared-decision making process and providing more avenues for person living with a rare disease (PLWRD) engagement and involvement at each stage of product design and development. Consequently, PLWRDs play a vital role in the implementation of new solutions that are ultimately designed to improve their own life and health.

The rapid evolution of genetic diagnostic techniques, including gene panels, whole exome, and whole genome sequencing, will provide new opportunities for early diagnosis of RDs potentially enabling PLWRDs to also have better access to therapies and treatments. Thus, genetic newborn screening (gNBS) could lead to a public health paradigm shift: early diagnosis, and intervention may prevent health damage before it is irreversible, decrease or avoid disability burden, and avoid premature death in potentially preventable diseases. Implementation of gNBS for rare diseases is thought to improve early access to care, redirect/focus treatment [7,8], impact pregnancy and family planning [9], and increase detection rate and accuracy of NBS [10].

However, undergoing gNBS has obvious ethical and social implications both for newborns and for parents as it may induce stress and increase concerns regarding diagnosis, treatment and the child´s future [11,12]. Additionally, there are challenges related to gNBS result interpretation, especially if based on whole-exome or whole-genome sequencing approaches,

For peer review only - http://bmjopen.bmj.com/site/about/guidelines.xhtml

communication of test results, occurrence of incidental findings and/or of variants of uncertain significance, data storage, and other issues that need to be addressed [13,14].

Bearing in mind that gNBS and the results of those tests may impact society and the individual itself, their adoption and implementation in medical practice depends on attitudes, experiences and preferences of different stakeholders. This study aims at understanding the general public's perception of gNBS and their preferences towards gNBS. Of particular interest are any differences in perceptions and preferences between medical geneticists, parents seeking genetic testing during their pregnancy and 'healthy' parents expecting a child.

Preference studies on genetic testing have been expanding, looking at various aspects and challenges from both patients and professionals' stakeholders [15].

Even if the general public attitude towards gNBS for rare diseases tends to be mostly positive [16-18], some research also pointed out that the acceptance of general public trends do not reflect the uptake of such testing during pregnancy [19]. The first part of our protocol will be a systematic literature review of preference study methods for gNBS (PROSPERO: CRD42022297678). Specific methods like discrete choice experiment (DCE) for eliciting preferences for gNBS have not been thoroughly implemented in Europe and only two previous DCEs studies in this field were identified. Miller et al. (2015) [20] assessed lay audiences' perception of gNBS in Canada. Respondents from the general public were positive about the potential for gNBS expansion in the country based on the expected clinical benefits, reproductive risk management improvement, and also the early diagnoses possibilities. Wright et al., (2018) [21], in another DCE, investigated information provision among midwives in the UK; when asking them about when to disclose information to expecting parents, or what type of information should be provided, they answered that "false positive" information should not be disclosed (non-clinically relevant), and that the best period to provide information was late pregnancy to 3 days post-birth. Performing a DCE, 7 years after the first DCEs on the topic in Europe [20] offers a comparison point for different cultural perspectives, attribute importance and technical advances in genetic techniques preferences. Moreover, this will allow us to assess the perspective of gNBS in Europe among expecting parents or individuals from general public who might actually face such a decision as we are on the verge of implementing such programs. In fact, most of the European countries that implemented neonatal screening in the 60s/ 70s are now implementing an extended panel, envisioning the potential to screen for 40 to 50 conditions to be tested with a single blood spot as a result of the technological improvements (molecular technologies) [22].

For peer review only - http://bmjopen.bmj.com/site/about/guidelines.xhtml

Our research will assess preferences from a more targeted population in comparison to general public. A recent international research protocol (6 countries) addressed the preferences of women toward prenatal testing results [23] which has the same stepwise structure as our protocol in a connected field (prenatal testing). From their first steps, based on a systematic literature review, they extracted 19 tentative attributes and refined them into 12. On the same prenatal topic, Buchanan et al. in 2022 [24] evaluated women's decision making with a DCE (12 scenarios) on prenatal genomic testing. These results were collected on an international (8 countries) level including a wide range of cultural examples from China to the USA, and showed that women looked for high diagnostic yield, short turnaround times and uncertain results to be reported (both variant of unknown significance and secondary findings). These attributes and choices may be considered for neonatal gNBS but they may also be different as the pregnancy planning decisions are not at stake anymore and the attributes of importance for new parents may be different after birth of their infant.

Methods and analysis

Screen4Care

This study is part of the larger project entitled 'Screen4Care (S4C)'. S4C is a research project launched in October 2021, which aims at shortening the diagnostic journey for rare diseases by accelerating diagnosis for PLWRDs through genetic newborn screening (gNBS) and digital technologies such as Artificial Intelligence (AI) and Machine Learning (ML). S4C has received funding from the Innovative Medicines Initiative 2 (IMI2) Joint Undertaking (JU). IMI is the world's largest public-private partnership in the life sciences whose mission is to "*improve health by speeding up the development of, and patient access to, innovative medicines, particularly in areas where there is an unmet medical or social need*".

Design of the study

The discrete choice experiment technique (DCE) will be applied in this study, which is a globally established technique to measure preferences in such areas as marketing, environmental economics, transportation, and health economics [25-28]. DCEs are based on Random Utility Theory[29-31]. Respondents are asked to complete several 'choice-tasks' (online), each of which consists of two or more alternatives that describe the treatment at hand. This description is based on treatment characteristics (i.e. attributes). It is assumed that the individual's preference for an alternative is determined based on the values of the attribute levels. Individuals' preferences can be inferred from the choices they make across multiple

For peer review only - http://bmjopen.bmj.com/site/about/guidelines.xhtml

choice-tasks. Using a DCE, researchers can quantify the importance of preferred treatment characteristics, calculate willingness-to-pay or willingness-to-accept risks and estimate potential participation rates, which makes this method highly attractive and superior to competing methodologies [25,26]. An example of a choice task for our study is included below in *Table 1*. The design and analysis of this survey will follow best practices [32,33].

Table 1. Example choice task DCE.

	Screening A	Screening B	No screening
Accuracy of screening test	90%	85%	40%
Waiting time between test and results	2 weeks	1 month	NA
Health care professional informing you about results	Nurse	GP	NA
I prefer:	0	0	0

Note: This figure is for illustrative purposes only. The final choice task will be determined after considering information learned from the interview and focus groups. A "no screening" alternative will be included in the DCE study since this option is necessary to estimate uptake of screening alternatives, including the proportion who would opt out of screening.

This prospective, non-interventional, cross-sectional study will be designed in a stepwise manner [28]. First, a systematic literature review will be performed to develop an initial assessment of the attitudes and perceptions of healthcare professionals and parents regarding gNBS for RDs. Data extraction forms were designed be used by researchers involved in this process. In order to ensure uniformity of data extraction, external reviewer from Lucid will provide a single reviewer analysis and quality assessment of the selected articles. Data will be analysed using thematic analysis. The main outcome of the review is a list of characteristics of gNBS that impact decisions of parents to participate in such screening or not (i.e., the list of potential attributes).

The qualitative research step will gather data using both focus groups and individual interviews. These two different types of interviews will collect both explorative and in-depth data. Focus groups will be conducted with expecting parents seeking genetic testing, while individual interviews will be conducted with *medical geneticists*. The individual interviews and focus groups are expected to start in 2023, and to be completed before June 2024. During all interviews a structured interview guide will be followed by trained interviewers. Before the interviews start, participants will be asked to sign an informed consent form (paper version if the group is held in person, or online form version if the group is held via video conference) and complete a short demographics survey (age, gender, number of previous pregnancies

For peer review only - http://bmjopen.bmj.com/site/about/guidelines.xhtml

(expecting parents only) and years of experience (clinicians only)). The focus groups and individual interviews will follow the structure of the nominal group technique (NGT; see [26]). After several introductory questions, participants will be asked to rank a list of potential attributes (emanating from the literature review) from most to least important according to their personal decision to take part in gNBS. These rankings will be discussed, and following the discussions participants will be allowed to change their ranking. Patient organisation representatives (members of the S4C Patients Advisory Board (PAB)who are patient representatives from 11 organisations who joined the consortium to ensure the PLWRDs' perspective is secured and prioritised in all research activities of Screen4Care) will review the short demographics survey and interview guides to share their input and ensure its understandability and appropriateness of the exact wording and definitions included in these documents.

Based on the attribute ranking in the qualitative portion of the study, the DCE will be designed. This DCE will be part of a larger survey including background questions and clinical scales (see *section Data Analysis*). The DCE itself will be constructed using a Bayesian D-efficient design with choice tasks consisting of two hypotheticals genetic new-born screening alternatives and a "no screening" alternative [25,34]. The educational section of the survey will be designed in close collaboration with patient organisation partners similarly to the interview guides, to ensure that the information is accurate and clear to all participants. Following development of the instrument the survey will be pretested amongst parents seeking genetic testing, "healthy" expecting parents, and medical geneticists to ensure they survey is understandable to the same population that will take the final online programmed survey in domestic languages. The members of the S4C PAB and Eurordis representatives will aid in drafting the survey and included training materials to assure readability and appropriateness of terms.

Objectives

To measure perspectives of medical geneticists, parents seeking genetic testing, and "healthy" expecting parents toward gNBS.

To elicit preferences of expecting parents from the general population as well as parents seeking genetic testing.

To identify and understand any heterogeneity in preferences based on demographic characteristics and attitudes towards screening and genetic testing in general.

For peer review only - http://bmjopen.bmj.com/site/about/guidelines.xhtml

To provide predicted uptake rates of gNBS based on the willingness to take part in rare disease screening program based on predefined characteristics of the screening test and output (not disease-specific).

Study Population and recruitment

For the qualitative part of this study three groups of stakeholders are identified: (1) medical geneticists (individual interviews), (2) expecting parents from the general population (focus groups), and (3) expecting parents currently seeking genetic testing (focus groups). These 3 groups of stakeholders will be recruited both in Germany and in Italy. All participants must be between the age of 18 and 70 years. Furthermore, they must be able to understand and speak German or Italian respectively as all interviews and survey material will be provided in native country-specific language (German or Italian).

Distribution of stakeholders group to study phases:

Medical geneticists are considered to be experts in precise genetic testing, advanced interpretation and results delivery to HCPs teams and patients. Medical geneticists involved in prenatal testing will be recruited via purposive sampling within clinical partners of Screen4Care project to be interviewed as experts.

The results of these interviews will not lead to any specific analyses but ensure the adequacy of the interview guide and questions.

The second group are "healthy" expecting parents from the general population (with pregnancy monitoring in regular care) recruited in the same geographic areas as the expecting parents currently seeking genetic testing for rare disease in one of the clinical centres. Focus group will be run by trained personal at the recruiting centre (trained midwifes, research nurses, depending on the available resources) that Uppsala University will support in the implementation and conduction of the groups. As a precautionary measure, if both partners volunteer to the study; the mother and father from the parental couple, will be included in separate groups to avoid any influence they may have on each other during the discussions. Recruitment of expecting parents from the general population will be conducted via midwifery and gynaecology practices in Germany and in Italy.

The third group of participants will include expecting parents who seek genetic testing but that are not necessarily carriers of genetic variants related to rare diseases themselves. They

For peer review only - http://bmjopen.bmj.com/site/about/guidelines.xhtml

will represent the expecting parents who are seeking genetic testing (having some concerns regarding genetic disease, excluding parents coming for preventive testing in the course of non-problematic pregnancy). Recruitment of expecting parents seeking genetic testing will be conducted by clinical genetic centres that are part of the Screen4Care project in both Germany and Italy. Patients will be identified as they visit the clinical centres for genetic testing purposes.

For the DCE survey expecting parents from the general population and expecting parents already seeking genetic testing will be recruited in Germany and Italy from S4C participating centers whereas parents from general population will be recruited from SurveyEngine participants lists. The study aims to recruit approximately 2084 participants in total testing across the two participating countries. For the qualitative phase, a maximum sample of 4 experts (two in Germany and two in Italy), 40 expecting parents (representing 20 couples in each country) for the group interviews will be recruited. The recruitment will be operated by the clinical centres and survey companies that have access to expecting parents from general population (total of 84 for the first part). For the quantitative phase, the survey sample will be about 1000 participants in each country (500 expecting parents from general population and 500 expecting parents seeking genetic testing) will be recruited from online Survey engine sample (general population) and from clinical centres (total of approximatively 2000 participants). A priori sample-size calculations represent a challenge in DCE experiments. Most published choice experiments have a sample size of 10 to more than 3000 respondents [35]. However, minimum sample size depends on several criteria, including the question format, the complexity of the choice task, the desired precision of the results, and the need to conduct subgroup analyses [32]. A method for computing sample size was proposed by de Bekker-Grob et al., 2015 [33], however, as the article points out there is no analytic solution or power calculation that can be used to determine the appropriate sample size for a DCE unless the researcher has enough information to inform the selection of priors. Additionally, for the general population sample we plan to use a blocked design (asking people to only complete a subset of the choice tasks, thereby limiting the burden on every single participant). Given what we know, recruiting 1000 respondents in each country should be sufficient to answer the proposed research questions and provide enough information to identify preferences in these groups and comparisons across these groups with acceptable precision.

Adapting Methods to Ethical specificities

For peer review only - http://bmjopen.bmj.com/site/about/guidelines.xhtml

As German, and Italian regulations may differ in their understanding of the risk level of focus group in terms of privacy and discomfort for the participants (e.g. genetic related conversation that could lead to sharing opinion or experience connected with genetics in front of other participants might be an argument for ethical committees' refusal in Italy). Methods could be adapted for example by conducting individual interviews for parents seeking genetic testing (more sensitive sample) and group interviews for general population expecting parents. Any informational material provided before signing the informed consent will point out the specific risks and expected benefits in order for participants to take an informed decision before entering the protocol.

Study Procedures

Information of participants and informed consent

Each participant will receive detailed written information on the nature, objectives, possible risks and benefits of the research (for both the focus groups and quantitative preference study participation). Prior to the qualitative interviews, participants will be given time to consider their involvement and ask questions about the study to the researchers. They will be informed that they can stop their participation in the study at each point. Participants can only take part in the study if informed consent form is signed beforehand. The investigators will keep the original signed informed consent in accordance to present requirements. For online survey participation, the informed consent will be included in the online survey and digitally signed by the participants.

For peer review only

Individual interviews to refine attribute selection and assign attribute levels

To select final attributes to be included in the DCE and to determine relevant attribute levels, we will organise several interviews with clinical experts and PLWRD's advocates. First, highest ranked attributes will be taken forward for consideration. For attributes to be included in the final DCE they should be unambiguous, not overlapping and clearly defined. Additionally, they should be important to the anticipated target population while at the same time clinically relevant. Therefore, the final selection of the most important attributes will be based on attribute ranking from the focus groups in combination with the perspective of expects

For peer review only - http://bmjopen.bmj.com/site/about/guidelines.xhtml

on practicalities of gNBS. After selecting the final attributes, appropriate levels that reflect current practice will inform the attributes list for the DCE.

These semi-structured interviews will be conducted with members of the IMI S4C consortium to test the interview guide and associated questions. These interviews will be conducted in English. The interview will take approximately one hour and will be conducted at a location convenient for the interviewee, via telephone, or via video conferencing platform. Feedback from the pilot interviews will be evaluated and the interview guides will be adapted accordingly. After making the necessary changes, the interview guide will be translated to German and Italian. Native language speaking trained interviewers from clinical centers will conduct the interviews using the interview guide. Collection, recording, and reporting of data will be will ensure the privacy, health, and welfare of research subjects during and after the study and in accordance with the country-specific regulations.

Group interviews

The group interviews will be used to provide more precise evidence for the most important characteristics of gNBS when parents decide about their participation in such screening programs. The information obtained from semi-structured individual interviews and the literature search will serve as a starting point for the development of the interview guide for the group interviews.

At the start of the interview the objective of the S4C project and the interview procedures will be explained to the interviewee and the researcher will confirm that informed consent was given before starting the interview. If informed consent was not given, the interviewee must sign the consent form before the start of the interview. Once informed consent is confirmed, the researcher will start the recording device. After asking the questions in the interview guide, participants will be asked to rank a list of potential attributes (emanating from the literature review) from most to least important according to their personal decision to take part in gNBS. These rankings will be discussed, and following the discussions participants will be allowed to change their ranking. The interviewer will ask the interviewees if they want to add something to the interview.

We plan to conduct a minimum of four group interviews per country (Germany, Italy) (see Figure 1). There will be eight group interviews, in total. A group interview consists of 8-10

For peer review only - http://bmjopen.bmj.com/site/about/guidelines.xhtml

members. The group interview will take about two hours and will be conducted at a location and on date most convenient for all participants.

The interviews will be recorded and transcribed verbatim. The interviews will be analysed iteratively using a grounded theory approach based on a mix of inductive and deductive coding. Analysis will be conducted in Atlas.ti software. The coding procedure will be handled by two independent coders from domestic countries. The outputs will be then translated into English for further use by the research team. To assure a proper standardisation of the interviews across languages, the domestic researchers will be trained by Uppsala's University researchers and will also be provided a detailed interview guide to guide them through the process of every interview.

The information given by the interviewee will be treated as confidential and will be processed anonymously. The collected data will be stored securely and viewed only by approved researchers of the project. The processed answers can be used for publication of reports or articles as part of the S4C research project.

The audio recordings specifically will be destroyed after completion of the study and no files will be stored at the recruiting centers.

Discrete Choice Experiment (DCE)

A survey will be fully developed after the final selection of attributes and levels. The survey instrument will also be pre-tested using think aloud methods in a convenience sample of 3 to 5 participants in each country using qualitative interviews to review the results from the interviews and to determine if any adjustments need to be made to improve clarity. This process will be conducted in collaboration with EURORDIS and patient organisation representatives' members of the PAB. They will ensure accurate use of vocabulary by assisting in drafting and reviewing the included definitions and explanatory texts as well as educational content to ensure accessibility and readability to lay audiences.

The initial DCE experimental design will be generated via NGene 1.0 (ChoiceMetrics, 2011) and hypothetical choice questions with three alternatives will be created (of which one is the 'no screening' alternative). These alternatives will be characterised by varying attribute levels. To ensure design efficiency, a pilot with 100 respondents in each country will be conducted. All participants will provide informed consent before entering the task. Based on

For peer review only - http://bmjopen.bmj.com/site/about/guidelines.xhtml

the data retrieved in the pilot test, a multinomial logit (MNL) model will be fitted. Beta estimates will be used to assign priors for the final experimental DCE design, which is a *d*-efficient (Bayesian) design [29] To increase efficiency of the design multiple blocks of choice tasks will be generated which will later be randomly distributed of respondents in such a way that each respondent will answer only one block of approximately 10-15 choice tasks.

Ethical and legal aspects

The global S4C IMI Project (grant agreement No. 101007757) has been approved by the Medical Ethics Committee of References. Ethical approval for the first part of the protocol has been approved in Germany, from University of Erlangen (22-246-B) the Freiburg University Ethics committee (23-1005-S1-AV) and in Italy (University of Ferrara: CE: 357/2023/Oss/AOUFe, and Hospedale Bambino Gesu: No.2997 of 2 November 2023, Prot. No. _902). Ethical Review Authorities in Sweden have approved the protocol for data storage and handling at Uppsala University (2022-05806-01).

The ethical procedure will be performed at two different stages, the first for the qualitative part and the second for the quantitative part (DCE). As the first part will be used as an input and will inform the second one, ethical committee may not consider approving a “tentative” survey draft or a “tentative” list of attributes as it can significantly change the ethical risk for anonymity (if one attribute is “cost” compare to “pregnancy planning”). The overall S4C project also ensured an ethical framework with the Code of Ethics Practice that was developed internally as a project Deliverable and agreed with all WP leaders in order to inform each researcher within the S4C consortium about the main ethical risks with no exception of this specific task on preferences studies.

Confidentiality

Due to the interaction of the researchers with the interviewees it is impossible to collect the data anonymously by recruiting centers but the obtained information will be coded to allow confidential and anonymous processing and reporting of the data from the research team. The identity of the participants will not be revealed so the privacy will be guaranteed. The research data can be used for publications in scientific journals and writing of reports or publication for the S4C project, but will be registered and saved with a secure code. The coded anonymous

For peer review only - http://bmjopen.bmj.com/site/about/guidelines.xhtml

data can be shared within the members of the consortium. The code, being the identification numbers linked to the identity of the participants, will be held confidential within the group of involved researchers. Only the researchers that are involved in this study will have access to this code and only they will know the identity of the participating stakeholder members.

Uppsala University will provide secure data storage space with restricted access to researcher analysing the data from the S4C partners involved in task 3.1. All digital data will be stored in Uppsala University's officially storage of research data (Dataportal Allvis) with specific ethical approval from The Swedish Ethical Review Authority, as Swedish Regulation requires. The data is physically stored in two redundant datacentres following (non-certified) ISO 27001 guidelines. Dataportal Allvis meets KRT value 233. Both datacentres are equipped with enterprise grade network, servers and hard disks with high availability, security and safety. All data centres are equipped with access controls, cooling systems, backup power and surge protection. They are protected against common threats such as fire, water damage, burglary and theft. Access is restricted to technical staff and strictly protocolled. All data is encrypted in transit (https) and at rest (through database encryption). Allvis is configured with versioning of files, saving up to 500 versions of an individual file. A recycle bin saves deleted files up to 30 days. Content databases are configured with transactional logging, limiting potential data losses to 1 hour during critical failures. Databases are backed up incrementally, daily, weekly, monthly and yearly using a TSM-based backup system hosted in the same data centers. Network traffic is protected through a robust firewall and servers hosting file content are not directly connected to the internet. The involved researchers commit to the highest standards of data security and protection in order to preserve the personal rights and interests of study participants. Participants will be informed that they can contact Uppsala University data security services for any information via email (dataskyddsombud@uu.se).

The involved researchers commit to the highest standards of data security and protection in order to preserve the personal rights and interests of study participants. They will adhere to the provisions set out in:

- The General Conference of the United Nations Educational, Scientific and Cultural Organization (UNESCO), meeting in Paris, from 9 to 24 November 2021, at its 41st session for Open science recommendations.
- A central guide for all research that includes human subjects will be the Declaration of Helsinki - Ethical principles for medical research involving human subjects from the

For peer review only - http://bmjopen.bmj.com/site/about/guidelines.xhtml

World Medical Association (WMA Adopted by the 18th WMA General Assembly, Helsinki, Finland, June 1964 -amended by the 64th WMA General Assembly, Fortaleza, Brazil, October 2013

- Directive 2002/58/EC of the European Parliament and of the Council of 12 July 2002 concerning the processing of personal data and the protection of privacy in the electronic communications sector (Directive on privacy and electronic communications)
- Regulation (EU) 2016/679 of the European Parliament and of the Council of 27 April 2016 on the protection of natural persons with regard to the processing of personal data and on the free movement of such data, and repealing Directive 95/46/EC (General Data Protection Regulation) (Text with EEA relevance)
- Regulation (EU) 2018/1725 of the European Parliament and of the Council of 23 October 2018 on the protection of natural persons with regard to the processing of personal data by the Union institutions, bodies, offices and agencies and on the free movement of such data, and repealing Regulation (EC) No 45/2001 and Decision No 1247/2002/EC

Data analysis

Based on the Systematic literature review, a list of tentative attributes will be extracted from Thematic analysis based on gNBS themes and recurrent factors but also coming from psychological influencing factors (e.g. depression, anxiety, emotion regulation) that will have to be addressed as contextual elements separately. As numerous elements are expected to be highlighted, the focus group will help clarify the most important elements. Independent coders will analyse the transcription of the focus group and provide content analysis. In order to refine the attribute list, the Ranking task will be performed presenting 15 attributes that may be reduced to 10 in order to design the DCE. The data from the ranking task will be analyzed to understand what attributes are most important to stakeholders. This information will be considered when deciding the final attributes for the DCE.

Participant characteristics that will be reported to describe the study samples include demographic characteristics (age, gender, pregnancy stage, income level among others), but also attitudes and psychosocial factors such as health literacy, and anxiety for example (Hospital Anxiety and Depression Scale, available in both Italian and German; Annunziata et al., 2011; Hinz & Brähler, 2011). In collaboration with recruiting centers we will consider the inclusion

For peer review only

For peer review only - http://bmjopen.bmj.com/site/about/guidelines.xhtml

of personality scales, emotion regulation scales and-or coping strategies scales to provide a more precise psychological assessment.

To determine preferences of study participants regarding gNBS, attribute (level) estimates and the conditional relative importance of attributes will be reported in the DCE using random parameters logit (RPL) modelling and latent class analyses (LCA). Heterogeneity of preferences and the impact of participant characteristics (e.g. demographics, psychological validated scales) will be investigated by applying appropriate statistical models including LCA for the DCE and/or subgroup analyses (RPL). All results described above will be compared between the German and Italian study sample to determine whether there are cross-country differences.

Finally, preferences and understanding of the survey instrument (comprehension questions) will be assessed by examining the relationship between psychological instruments of cognitive ability (e.g. health literacy and numeracy) and survey responses. This includes comparing responses to warm up questions, survey internal validity checks, and attitudinal questions about reported complexity of tasks across different levels of each psychological instrument.

For peer review only

For peer review only - http://bmjopen.bmj.com/site/about/guidelines.xhtml

Acknowledgements:

Author contributions: Sylvia Martin (SM), Joshua Coulter (JC), Jorien Veldwijk (JV) drafted the protocol and closely ensures the adaptation of the final version to all authors's comments.

Sylvia Martin (SM), Joshua Coulter (JC), Jorien Veldwijk (JV) Åsa Grauman (AG), Brett Hauber (BH), Mats Hansson (MH), contributed to the design of the protocol.

Ferdinand Knieling (FK), Roman Raming (RR), Janbernd Kirchner (JK), Stefaan Sansen (SS), Christina Saier (CS), Lucia Pia Bruno (LPB), Vera Frankova (VF), Fernanda Fortunato (FF), Alessandra Ferlini (AF), Nicolas Garnier (NG), Antonio Novelli (AN), Emanuele Angolini (EA), Enrico Silvio Bertini (ESB), and Silvia Ottombrino (SO) drafted the precise clinical information used in the draft, checked for relevance of the vocabulary and reviewed the overall structure of the article.

Non-authors contribution: We thank the members of the Screen 4 Care Patient advisory board for their constructive help during consultation meeting and for the quality of their written feedbacks.

Funding statement: 'This work was supported by Sreen4Care. This project has received funding from the Innovative Medicines Initiative 2 Joint Undertaking (JU) under grant agreement No 101034427. The JU receives support from the European Union's Horizon 2020 research and innovation programme and EFPIA.

Competing interests' statement: all authors filled in the coi-disclosure forms. As some authors changed affiliation during after participating their affiliations will be stated as they were at the time of the writing of the present article. Any question about contacting a co-author of the protocol can be send to sylvia.martin@crb.uu.se .

Data statement: Data will be made available under reasonable demand to the corresponding authors and in accordance to Screen 4 Care data security procedures.

For peer review only - http://bmjopen.bmj.com/site/about/guidelines.xhtml

References:

[1] Nguengang Wakap, S., Lambert, D. M., Olry, A., Rodwell, C., Gueydan, C., Lanneau, V., Murphy, D., Le Cam, Y., & Rath, A. (2020). Estimating cumulative point prevalence of rare diseases: analysis of the Orphanet database. *European journal of human genetics : EJHG*, *28*(2), 165–173. https://doi.org/10.1038/s41431-019-0508-0

[2] Hay E, Elmslie F, Lanyon P and Cole T. The Diagnostic Odyssey in rare diseases; a Task and Finish Group report for the Department of Health and Social Care [version 1; not peer reviewed]. *NIHR Open Res* 2022, **2**:3 (document) (https://doi.org/10.3310/nihropenres.1115171.1)

[3] Marwaha, S., Knowles, J. W., & Ashley, E. A. (2022). A guide for the diagnosis of rare and undiagnosed disease: beyond the exome. *Genome medicine*, *14*(1), 1-22.

[4]Ferreira C. R. (2019). The burden of rare diseases. *American journal of medical genetics. Part A*, *179*(6), 885–892. https://doi.org/10.1002/ajmg.a.61124

[5] Gokdemir, Y., Eyuboglu, T. S., Emiralioglu, N., Er, B., Sen, V., Pekcan, S., ... & Yılmaz, O. (2021). Geographical barriers to timely diagnosis of cystic fibrosis and anxiety level of parents during newborn screening in Turkey. *Pediatric Pulmonology*, *56*(10), 3223-3231.

[6]Grob, R., Roberts, S., & Timmermans, S. (2018). Families' Experiences with Newborn Screening: A Critical Source of Evidence. *Hastings Center Report*, *48*, S29-S31.

[7] Bailey Jr, D. B., Bishop, E., Raspa, M., & Skinner, D. (2012). Caregiver opinions about fragile X population screening. *Genetics in Medicine*, *14*(1), 115-121.

[8] van Dijk, T., Kater, A., Jansen, M., Dondorp, W. J., Blom, M., Kemp, S., ... & Henneman, L. (2021). Expanding Neonatal Bloodspot Screening: A Multi-Stakeholder Perspective. *Frontiers in pediatrics*, *9*, 706394.

[9] Chavez-Yenter, D., Vagher, J., Clayton, M. F., Rindler, M., Shukovich, M., & Kaphingst, K. A. (2021). "Being proactive, not reactive": exploring perceptions of genetic testing among White, Latinx, and Pacific Islander Populations. *Journal of Community Genetics*, *12*(4), 617-630.

[10] Perobelli, S., Zanolla, L., Tamanini, A., Rizzotti, P., Maurice Assael, B., & Castellani, C. (2009). Inconclusive Cystic Fibrosis neonatal screening results: long-term psychosocial effects on parents. *Acta Paediatrica*, *98*(12), 1927-1934.

[11] Pruniski, B., Lisi, E., & Ali, N. (2018). Newborn screening for Pompe disease: Impact on families. *Journal of inherited metabolic disease*, *41*(6), 1189-1203.

[12] Bailey Jr, D. B., Berry-Kravis, E., Gane, L. W., Guarda, S., Hagerman, R., Powell, C. M., ... & Wheeler, A. (2017). Fragile X newborn screening: lessons learned from a multisite screening study. *Pediatrics*, *139*(Supplement_3), S216-S225.

[13] Friedman, J.M., Cornel, M.C., Goldenberg, A.J. *et al.* Genomic newborn screening: public health policy considerations and recommendations. *BMC Med Genomics* **10**, 9 (2017). https://doi.org/10.1186/s12920-017-0247-4

[14] Knoppers, B. M., Sénécal, K., Borry, P., & Avard, D. (2014). Whole-genome sequencing in newborn screening programs. *Science translational medicine*, *6*(229), 229cm2-229cm2. DOI: 10.1126/scitranslmed.3008494

For peer review only - http://bmjopen.bmj.com/site/about/guidelines.xhtml

[15] Ozdemir, S., Finkelstein, E., Lee, J. J., Too, I. H. K., Teo, K. Y. C., Tan, A. C. S., ... & Cheung, G. C. M. (2022). Understanding patient preferences in anti-VEGF treatment options for age-related macular degeneration. *PloS one*, *17*(8), e0272301.

[16] Moultrie, R. R., Paquin, R., Rini, C., Roche, M. I., Berg, J. S., Powell, C. M., & Lewis, M. A. (2020). Parental views on newborn next generation sequencing: implications for decision support. *Maternal and child health journal*, *24*, 856-864.

[17] Bombard, Y., Miller, F. A., Hayeems, R. Z., Barg, C., Cressman, C., Carroll, J. C., ... & Chakraborty, P. (2014). Public views on participating in newborn screening using genome sequencing. *European Journal of Human Genetics*, *22*(11), 1248-1254.

[18] Plass, A. M. C., van El, C. G., Pieters, T., & Cornel, M. C. (2010). Neonatal screening for treatable and untreatable disorders: prospective parents' opinions. *Pediatrics*, *125*(1), e99-e106.

[19] Bailey, D. B., Lewis, M. A., Harris, S. L., Grant, T., Bann, C., Bishop, E., ... & Therrell, B. L. (2013). Design and evaluation of a decision aid for inviting parents to participate in a fragile X newborn screening pilot study. *Journal of genetic counseling*, *22*(1), 108-117.

[20] Miller, F. A., Hayeems, R. Z., Bombard, Y., Cressman, C., Barg, C. J., Carroll, J. C., ... & Regier, D. A. (2015). Public perceptions of the benefits and risks of newborn screening. Pediatrics, 136(2), e413-e423.

[21] Wright, S. J., Ulph, F., Lavender, T., Dharni, N., & Payne, K. (2018). Understanding midwives' preferences for providing information about newborn bloodspot screening. *MDM policy & practice*, *3*(1), 2381468317746170.

[22] Loeber, J. G., Platis, D., Zetterström, R. H., Almashanu, S., Boemer, F., Bonham, J. R., Borde, P., et al. (2021). Neonatal Screening in Europe Revisited: An ISNS Perspective on the Current State and Developments Since 2010. *International Journal of Neonatal Screening*, *7*(1), 15. MDPI AG. Retrieved from http://dx.doi.org/10.3390/ijns7010015

[23]Hammond, J., Klapwijk, J. E., Riedijk, S., Lou, S., Ormond, K. E., Vogel, I., ... & Lewis, C. (2022). Assessing women's preferences towards tests that may reveal uncertain results from prenatal genomic testing: Development of attributes for a discrete choice experiment, using a mixed-methods design. *PloS one*, *17*(1), e0261898.

[24] Buchanan, J., Hill, M., Vass, C. M., Hammond, J., Riedijk, S., Klapwijk, J. E., ... & Lewis, C. (2022). Factors that impact on women's decision-making around prenatal genomic tests: An international discrete choice survey. *Prenatal diagnosis*, *42*(7), 934-946.

[25] Hensher, D., J.M. Rose, and W.H. Greene, *Applied Choice Analysis: second edition*. 2015, Cambridge, UK: Cambridge University Press.'

[26] Hiligsmann, M., et al., Nominal group technique to select attributes for discrete choice experiments: an example for drug treatment choice in osteoporosis. Patient Prefer Adherence, 2013; 7:133-9

[27] Reed Johnson, F., E. Lancsar, D. Marshall, V. Kilambi, A. Muhlbacher, D.A. Regier, B.W. Bresnahan, B. Kanninen, and J.F. Bridges, Constructing experimental designs for discrete-choice experiments: report of the ISPOR Conjoint Analysis Experimental Design Good Research Practices Task Force. *Value Health*, 2013. 16(1): p. 3-13.

[28] Bridges, J.F., A.B. Hauber, D. Marshall, A. Lloyd, L.A. Prosser, D.A. Regier, F.R. Johnson, and J. Mauskopf, Conjoint analysis applications in health--a checklist: a report of the

For peer review only - http://bmjopen.bmj.com/site/about/guidelines.xhtml

ISPOR Good Research Practices for Conjoint Analysis Task Force. *Value Health*, 2011. 14(4): p. 403-13.

[29] Kessels, R., et al., An efficient algorithm for constructing bayesian optimal choice designs. Journal of Business & Economic Statistics, 2009; 27(2):279-291.

[30] Louviere, J.J., et al., Designing discrete choice experiments: do optimal designs come at a price? Journal of Consumer Research, 2008; 35(2):360-375.

[31] Marshall, D., et al., Conjoint Analysis Applications in Health - How are Studies being Designed and Reported?: An Update on Current Practice in the Published Literature between 2005 and 2008. Patient., 2010; 3(4):249-56.

[32] Louviere, J., D. Hensher, and J. Swait, Stated Choice Methods; analysis and application.. 2000, Cambridge: Cambridge University Press.

[33]de Bekker-Grob, E.W., B. Donkers, M.F. Jonker, and E.A. Stolk, Sample Size Requirements for Discrete-Choice Experiments in Healthcare: a Practical Guide. *Patient*, 2015. 8(5): p. 373-84.

[34] Rose, J. M., & Bliemer, M. J. C. (2009). Constructing efficient stated choice experimental desings. *Transport Reviews, 9, 1–31*.

[35] Merlo, G., van Driel, M., & Hall, L. (2020). Systematic review and validity assessment of methods used in discrete choice experiments of primary healthcare professionals. *Health economics review*, *10*(1), 1-9.

For peer review only

BMJ Open

Patient Preferences in Genetic Newborn Screening for Rare diseases: study protocol

Journal:	*BMJ Open*
Manuscript ID	bmjopen-2023-081835.R1
Article Type:	Protocol
Date Submitted by the Author:	28-Jan-2024
Complete List of Authors:	MARTIN, Sylvia; Uppsala Universitet, Center for Research and Bioethics<br>Angolini, Emanuele; Ospedale Pediatrico Bambino Gesù, Research Unit of Neuromuscular and Neurodegenerative Disease<br>Audi, Jennifer; Takeda Pharmaceuticals International AG<br>Bertini, Dr. Enrico; Ospedale Pediatrico Bambino Gesù, Research Unit of Neuromuscular and Neurodegenerative Disease<br>Bruno, Lucia Pia; University of Siena, Medical Genetics; Telethon Institute of Genetics and Medicine<br>Coulter, Joshua; Pfizer Global Pharmaceuticals<br>Ferlini, Alessandra; University of Ferrara, Department of Medical Sciences<br>Fortunato, Fernanda; University of Ferrara, Department of Medical Sciences<br>Frankova, Vera; Charles University First Faculty of Medicine<br>Garnier, Nicolas; Pfizer Inc<br>Grauman, Åsa; Uppsala Universitet, Center for Research and Bioethics<br>Gross, Edith; EURORDIS<br>Hauber, Brett; Pfizer Inc<br>Hansson, Mats; Uppsala Universitet, Center for Research and Bioethics<br>Kirschner, Janbernd; University of Freiburg<br>Knieling, Ferdinand; Erlangen University Hospital<br>Kyosovksa, Gergana; Bulgarian Association for Personalised Medicine<br>Ottombrino, Silvia; Ospedale Pediatrico Bambino Gesù, Research Unit of Neuromuscular and Neurodegenerative Disease<br>Novelli, Antonio; Ospedale Pediatrico Bambino Gesù, Research Unit of Neuromuscular and Neurodegenerative Disease<br>Raming, Roman; Erlangen University Hospital<br>Sansen, Stefaan; Sanofi-Aventis SA, Department of Medical Biotechnologies<br>Saier, Christina; Faculty of Medicine, Department of Neuropediatrics and Muscle Disorders<br>Veldwijk, Jorien; Erasmus Universiteit Rotterdam
**Primary Subject Heading**:	Genetics and genomics
Secondary Subject Heading:	Qualitative research, Research methods
Keywords:	GENETICS, QUALITATIVE RESEARCH, Patient-Centered Care, Patient Participation, Patient Reported Outcome Measures

For peer review only - http://bmjopen.bmj.com/site/about/guidelines.xhtml

SCHOLARONE™
Manuscripts

Patient Preferences in Genetic Newborn Screening for Rare diseases: study protocol

Authors: Sylvia Martin[1*], Angolini Emanuele[14], Jennifer Audi[5], Enrico Silvio Bertini[14], Lucia Pia Bruno[7,8], Joshua Coulter[3], Alessandra Ferlini[12], Fernanda Fortunato[12], Vera Frankova[9], Nicolas Garnier[3], Åsa Grauman[1], Edith Gross[13], Brett Hauber[3], Mats Hansson[1.], Janbernd Kirschner[10], Ferdinand Knieling[6], Gergana Kyosovksa[4], Silvia Ottombrino[14], Antonio Novelli[14], Roman Raming[6], Stefaan Sansen[8], Christina Saier[10], Jorien Veldwijk [1,2].

For peer review only

Word count: 5933

Funding: Sreen4Care project, Innovative Medicines Initiative grant agreement No 101034427.

1. Centre for Research and Bioethics (CRB), Uppsala University, Sweden, Husargatan 3, BMC, entrance A11.

2. Erasmus School of Health Policy & Management, Erasmus University Rotterdam, P.O.Box 1738, 3000 DR Rotterdam, The Netherlands.

3. Pfizer, 235 E 42nd St, New York, USA.

4. Bulgarian Association for Personalised Medicine, 45 Bacho Kiro str., 1202 Sofia, Bulgaria.

5. Takeda Pharmaceuticals International AG, Zurich, Switzerland

6. Departmenf of Pediatrics and Adolescent Medicine, Universitätsklinikum Erlangen, Friedrich-Alexander-Universität (FAU) Erlangen-Nürnberg, Erlangen, Germany.

7. Medical Genetics, University of Siena, Siena, Italy

8. Med Biotech Hub and Competence Center, Department of Medical Biotechnologies, University of Siena, Siena, Italy.

9. Institute for Medical Humanities, Charles University First Faculty of Medicine, Karlovo náměstí 40, 128 00 Prague 2, Czech Republic

10. Department of Neuropediatrics and Muscle Disorders, Medical Center – University of Freiburg, Faculty of Medicine, Freiburg, Germany

11. Sanofi, Diegem, Belgium.

12. Unit of Medical Genetics, Department of Medical Sciences, University of Ferrara, Via Fossato di Mortara, 74, 40122 Ferrara (Italy)

13. EURORDIS, Plateforme Maladies Rares,96, rue Didot, 75014 Paris (France).

14. Research Unit of Neuromuscular and Neurodegenerative Disease, Ospedale Pediatrico Bambino Gesu, Roma, Lazio (Italy).

* Sylvia Martin is the corresponding author: Centre for Research and Bioethics (CRB), Uppsala University, Sweden, Husargatan 3, BMC, entrance A11. sylvia.martin@crb.uu.se

For peer review only

Abstract:

Introduction: Rare diseases collectively impact over 30 million people in Europe. Most individual conditions have a low prevalence which has resulted in a lack of research and expertise in this field, especially regarding genetic newborn screening (gNBS). There is increasing recognition of the importance of incorporating patients' needs and general public perspectives into the shared-decision making process regarding gNBS. This study is part of the Innovative Medicine Initiative project Screen 4 Care which aims at shortening the diagnostic journey for rare diseases by accelerating diagnosis for patient living with rare diseases through genetic newborn screening (gNBS) and the use of digital technologies, such as Artificial Intelligence (AI) and Machine Learning (ML).

Objective: Assessing expecting parent's perspectives, attitudes and preferences regarding gNBS for rare diseases in Italy and Germany.

Methods and analysis: A mixed method approach will assess perspectives, attitudes and preferences of (1) expecting parents seeking genetic consultation and (2) "healthy" expecting parents from general population in two countries (Germany and Italy). Focus groups and interviews using the nominal group technique and ranking exercises will be performed (qualitative phase). The results will inform the treatment of attributes to be assessed via a survey and a discrete choice experiment (DCE). The total recruitment sample will be 2084 participants (approximatively 1000 participants in each country for the online survey). A combination of thematic qualitative and logit-based quantitative approaches will be used to analyze the results of the study.

Ethics and dissemination: This study has been approved by the Erlangen University Ethics Committee (22-246_1-B), the Freiburg University Ethics committee (23-1005-S1-AV) and for clinical centers in Italy (University of FerraraCE: 357/2023/Oss/AOUFe, and Hospedale Bambino Gesu: No.2997 of 2 November 2023, Prot. No. _902), and approved for data storage and handling at Uppsala University (2022-05806-01). The dissemination of the results will be ensured via scientific journal publication (Open Access).

Strengths and limitations of this study:

- This study has been developed by an international expert team together with industry and patient representatives to provide an example of a rigorously designed 2-stepped preferences study.

For peer review only

-This study will collect data from a significantly large sample across several populations, allowing comparison of gNBS uptake perspectives, attitudes and preferences, psychological variables, and socio-demographic information in two European countries.

- Only the most important characteristics of gNBS can be incorporated into the DCE, therefore considerations of less important characteristics can only be interpreted based on the outcomes of the qualitative work and have to be assumed less important (on average) than the least important outcome of the DCE.

-Study elements might be complex (cross cultural elements, potential issues in translations, recruitment populations differences) and prone to misunderstanding even if high involvement of patient representatives in the development of the study material will reduce these risks.

-This study will recruit participants from different countries where the in-place gNBS procedure is differently implemented in the general care settings.

Keywords: genetic newborn screening, rare disease, patient preference, discrete choice experiment.

For peer review only

Introduction

There are more than 7,000 known rare diseases (RDs), conditions that affect one, or less than one, individual in 2,000 (point prevalence of <1/1 000 000 in more than 84% of RDs [1]). People living with rare a disease (PLWRD[1]) typically face an arduous journey to proper diagnosis, enduring on average eight years of countless physician consultations [2]. Time to diagnosis is a stressful period during which patients are faced with inconclusive test outcomes, possible misdiagnoses, and thereby ineffective treatments and healthcare resource utilisation [3].

Although RDs collectively impact over 30 million people in Europe, most individual conditions have a low prevalence [4]. This has resulted in a lack of research and expertise in this field. Difficulties in the process of receiving an accurate diagnosis and uncertainty around experienced symptoms continue to add to the difficulties that PLWRD experience, but also affecting their families, caregivers, physicians, and society as a whole [5,6]. Currently, there is increased recognition of the importance of incorporating patients' needs and perspectives into the shared decision-making process and providing more avenues for engagement and involvement of PLWRD at each stage of the design and development of health interventions. Consequently, PLWRDs play a vital role in the implementation of novel solutions that are designed to improve their own life and health.

The rapid evolution of genetic diagnostic techniques, including gene panels, whole exome, and whole genome sequencing, will provide new opportunities for early diagnosis of RDs potentially enabling PLWRDs to also have better access to therapies and treatments. Thus, genetic newborn screening (gNBS)[2] could lead to a public health paradigm shift with early diagnosis and intervention which may prevent health damage before it is irreversible and avoid functional limitations leading to premature death in potentially preventable diseases.

---

[1] This acronym has been selected based on its usage in international literature https://www.rarediseasesinternational.org/wp-content/uploads/2022/01/Final-UN-Text-UN-Resolution-on-Persons-Living-with-a-Rare-Disease-and-their-Families.pdf

[2] We will use a broad definition of genetic newborn screening as defined by Institute NHGR. 2023, 17th November [Available from: https://www.genome.gov/genetics-glossary/Newborn-Genetic-Screening : "*Newborn screening is a set of laboratory tests performed on newborn babies to detect a set of known genetic diseases. Typically, this testing is performed on a blood sample obtained from a heel prick when the baby is two or three days old. […].*"

For peer review only

Implementation of gNBS for RDs is thought to improve early access to care, redirect/focus treatment [7,8], impact pregnancy and family planning [9], and increase detection rate and accuracy of newborn screening programs as a whole [10].

However, undergoing gNBS has obvious ethical and social implications both for newborns and for parents as it may induce stress and increase concerns regarding diagnosis, treatment, and the child´s future [11,12]. Ethical and psycho-social issues related to gNBS present considerable dilemmas regarding the effects of genetic data on both the infant and their family, as well as with regard to broader societal aspects. These concerns play a critical role in shaping regulatory structures and health policies aimed at addressing these ethical obstacles (Reinsteing, 2015; Grob, 2019). The National Human Genome Research Institute *Newborn Screening Fact Sheet* states, *"with the decreasing cost of genome sequencing, there is potential for its clinical application in newborn screening. This could supplement or replace traditional panels of tests, providing more comprehensive health information. However, several questions remain about the practicality, ethics, and long-term implications of incorporating genome sequencing into routine newborn screening*" (https://www.genome.gov/about-genomics/fact-sheets/Newborn-Screening-Fact-Sheet, consulted on January 2 2024). Therefore, clarifying HCPs', patients', and general public perspectives, attitudes and preferences on gNBS implications may improve its safe and equitable development.

Additionally, there are challenges related to interpretation of gNBS results, especially if based on whole-exome or whole-genome sequencing approaches: communication of test results, management of incidental findings and/or of variants of uncertain significance, data storage, and other issues that need to be addressed [13,14].

Bearing in mind that gNBS and its results may impact society and the individual itself, their adoption and implementation in medical practice depends on attitudes, experiences, and preferences of different stakeholders. This study aims to understand the general public's perspectives and attitudes of gNBS and their preferences towards gNBS. Of particular interest are any differences in perspectives, attitudes and preferences between medical geneticists, expecting parents seeking genetic consultation during their pregnancy, and 'healthy' expecting parents.

There has been an increase in preference studies on genetic screening. These studies examine various aspects and challenges from both patients and health care professional stakeholders [15]. Even if the general public's attitude towards gNBS for RDs tends to be

For peer review only

positive [16-18], research also demonstrated that the acceptance of gNBS in the general public does not reflect the uptake of gNBS during pregnancy [19].

The first part of our protocol describes a systematic literature review of preference study results for gNBS (PROSPERO: CRD42022297678) in order to inform the design of the qualitative and quantitative parts of the protocol. Specific methods like discrete choice experiment (DCE) for eliciting preferences for gNBS have not been thoroughly implemented in Europe and only two previous DCEs studies in this field were identified. Miller et al. (2015) [20] assessed the perception of lay audiences regarding gNBS in Canada. Respondents from the general public were positive about the potential for gNBS expansion in the country based on the expected clinical benefits, improvement of reproductive risk management, and also the possibility of earlier diagnosis. Wright et al., (2018) [21], in another DCE, investigated information provision among midwives in the UK; when asking them about when to disclose information to expecting parents, or what type of information should be provided. According to this study, the potential for receiving a "false positive" result should not be disclosed (as it may not be helping decision making), and the best period to provide information would be late pregnancy to 3 days post-birth. The current study protocol, 7 years after the first DCEs on the topic in Europe [20], offers a comparison point for diverse cultural perspectives, attribute importance and technical advances in genetic techniques preferences. Moreover, this study will allow us to assess the perspective of gNBS in Europe among expecting parents who might face such a decision as health care systems are on the verge of implementing or extending gNBS programs. In fact, most of the European countries that implemented neonatal screening in the 60s/ 70s are now implementing an extended panel, envisioning the potential to screen for 40 to 50 conditions to be tested with a single blood spot using improvements in molecular technologies [22]. Protocols for gNBS research offer innovations for prompt and efficient treatment, as well as reducing obstacles related to worry in parents or over diagnosis (Gray et al., 2008). The research about gNBS serves as a catalyst for ongoing advancements in screening's sensitivity and specificity. The goal of the screening process optimization efforts is to provide assurance that true positive cases will be accurately diagnosed and to prevent families from being upset by an unexpected false positive result or from experiencing severe anxiety as an effect of results with uncertain significance (La Marca et al., 2023). This study will inform both clinical research and patient advocates stakeholders contributing to gNBS for RDs field.

For peer review only

A recent international research protocol (6 countries) addressed the preferences of women toward prenatal screening results [23], with the same stepwise structure as our protocol but in a connected field (prenatal testing). Based on a systematic literature review, they extracted 19 tentative attributes and refined them into 12. On the same prenatal topic, Buchanan et al. in 2022 [24] evaluated womens' decision making on prenatal genomic screening with a DCE (12 scenarios). These results were collected on an international (8 countries) level including a wide range of cultural examples from China to the United States, and showed that women looked for high diagnostic yield, short turnaround times and uncertain results to be reported (both variant of unknown significance and secondary findings).

These attributes and choices may be considered for neonatal gNBS but they may be different as the pregnancy planning decisions are not at stake anymore and the attributes of importance for new parents may be different after birth of their infant. Our research will assess the perspectives, attitudes and preferences from a more targeted population of expecting parents.

Methods and analysis

Research Aims and Objectives: This study is part of the larger project entitled 'Screen4Care (S4C)'. S4C is a research project launched in October 2021, which aims at shortening the diagnostic journey for rare diseases by accelerating diagnosis for PLWRDs through gNBS and digital technologies such as Artificial Intelligence (AI) and Machine Learning (ML). S4C has received funding from the Innovative Medicines Initiative 2 (IMI2) Joint Undertaking (JU). IMI, now superseded by IHI (Innovative Health Initiatives), is the world's largest public-private partnership in the life sciences whose mission is to "*improve health by speeding up the development of, and patient access to, innovative medicines, particularly in areas where there is an unmet medical or social need.*"

The expected start of the study is December 2023 for a planned end in July 2024. The study has several objectives, being:

- To measure perspectives and attitudes of medical geneticists, parents seeking genetic consultation, and "healthy" expecting parents for gNBS.
- To elicit preferences of "healthy" expecting parents from the general population as well as of parents seeking genetic consultation.
- To identify and understand any heterogeneity in preferences for gNBS based on demographic characteristics and attitudes towards screening and genetic testing in general.

For peer review only

- To provide predicted uptake rates of gNBS based on the willingness to take part in rare disease screening program based on predefined characteristics of the screening test and output (not disease-specific).

Patient and Public involvement: Research questions and information materials have been developed with patient representatives.

Design of the study

A discrete choice experiment technique (DCE) will be used in this study, which is a globally established technique to elicit preferences in areas such as marketing, environmental economics, transportation, and health economics [25-28]. DCEs are based on Random Utility Theory [29-31]. Respondents are asked to complete several 'choice-tasks' (online), each of which consists of two or more alternatives that describe a treatment at hand. This description is based on treatment characteristics (i.e. attributes). It is assumed that an individual's preference for an alternative is based on the values of the attribute levels. Individuals' preferences can be inferred from the choices made across multiple choice-tasks. Using a DCE, researchers can quantify the importance of preferred treatment characteristics, calculate willingness-to-pay or willingness-to-accept risks and estimate potential participation rates, which makes this method highly attractive and superior to competing methodologies [25,26]. An example of a choice task for our study is included below in *Table 1*. The design and analysis of this survey will follow best practices [32,33]. Since a DCE can only include a total of about 5-7 of the most important attributes, it will not provide a fully holistic overview of the importance of all characteristics of gNBS on the decision to participate in such a screening procedure. However, using only the most important attributes results, uptake behaviour can be correctly predicted as the positive predictive value of DCEs has been shown to be over 0.90 (De Bekker-Grob et al., 2020)

Table 1. Example choice task DCE.

	Screening A	Screening B	No screening
Accuracy of screening test	90%	85%	Not applicable
Waiting time between test and results	2 weeks	1 month	NA

For peer review only - http://bmjopen.bmj.com/site/about/guidelines.xhtml

Health care professional informing you about results	Nurse	General Practitioner	NA
I prefer:	0	0	0

Note: This figure is for illustrative purposes only. The final choice task will be determined after considering information learned from the interview and focus groups. A "no screening" alternative will be included in the DCE study since this option is necessary to estimate uptake of screening alternatives, including the proportion who would opt out of screening.

This prospective, non-interventional, cross-sectional study will be designed in a stepwise manner [28]. First, a systematic literature review will be performed to develop an initial assessment of the perspectives and attitudes of healthcare professionals and parents regarding gNBS for RDs. Data extraction forms were designed by researchers involved in this process (11 duos of experts from the S4C project). Following the initial selection, an external firm specializing in various databases and JBI tools, LUCID ltd, located in Marlow, England, conducted a scientific quality evaluation (appraisal scoring). Subsequently, only those articles that exhibited a quality level above the average in their respective category were selected for further analysis. Data was analysed using thematic analysis. The main outcome of the review was a list of characteristics of gNBS that impact decisions of parents to participate in such screening or not (i.e., the list of potential attributes)[3].

The qualitative research step will gather data using both focus groups and individual interviews. These two different types of interviews will collect both explorative and in-depth data. Focus groups will be conducted with expecting parents seeking genetic consultation as well as "healthy" expecting parents, while individual interviews will be conducted with medical geneticists. The individual interviews and focus groups are expected to start in 2023, and to be completed before June 2024. During all interviews, a structured interview guide will be followed by trained interviewers. Before the start of the interviews, participants will be asked to sign an informed consent form (paper version if the group is held in person, or online form version if the group is held via video conference) and complete a short demographics survey (age, gender, number of previous pregnancies (expecting parents only) and years of experience (clinicians only)). The focus groups and individual interviews will follow the structure of the nominal group technique (NGT; see [26]). After several introductory questions, participants

[3] The output of the systematic literature review is planned to be published separately as a stand-alone paper in a scientific journal.

will be asked to rank a list of potential attributes (emanating from the literature review) from most to least important according to their personal willingness to take part in gNBS. These rankings will be discussed, and, following the discussions, participants will be allowed to change their ranking. Patient representatives from 11 organizations, who are a member of the S4C Patients Advisory Board (PAB) and joined the consortium to ensure that the perspective of PLWRDs is secured and given priority in all research activities of Screen4Care, will review the interview guides and short demographics survey to provide feedback and make sure the wording and definitions used are understandable and appropriate.

Based on the attribute ranking in the qualitative portion of the study, the DCE will be designed. This DCE will be part of a larger survey including background questions and clinical scales (see *section Data Analysis*). Given the number of attributes and levels included in the DCE, respondents would not be able to complete a full factorial design (i.e., over 1000 choice tasks). Most DCEs have respondents answer 8-15 choice questions. Blocked designs offer the opportunity to increase the efficiency of the data collection while the cognitive load on respondents stays low ass they only complete a subsection (block) of the total number of choice tasks. Therefore, such designs are typical in DCEs (Johnson et al., 2013). When blocking a design, all respondents will still see all attributes and levels but only a subset of combinations. The blocks of questions each respondent receives will be randomized to mitigate any potential bias of the blocked design while maximizing statistical power of the full design in the total study population (Hensher et al., 2015).

The DCE itself will be constructed using a Bayesian D-efficient design with choice tasks consisting of two hypothetic gNBS alternatives and a "no screening" alternative [25,34]. The educational section of the survey will be designed in close collaboration with patient organisation partners (PAB and EURORDIS representatives) similarly to the interview guides, to ensure that the information is accurate and clear to all participants, and assuring readability and appropriateness of terms. Following development of the instrument the survey will be pretested amongst expecting parents seeking genetic consultation, "healthy" expecting parents, and medical geneticists to ensure that the survey is understandable to the same population that will take the final online programmed survey in domestic languages.

Study Population and recruitment

For the qualitative part of this study three groups of interviewees are identified: (1) medical geneticists (individual interviews), (2) "healthy" expecting parents from the general population

(focus groups), and (3) expecting parents currently seeking genetic consultation (focus groups). These 3 groups will be recruited both in Germany and in Italy. All participants must be between the age of 18 and 70 years. Furthermore, they must be able to understand and speak German or Italian respectively as all interviews and survey material will be provided in native country-specific language (German or Italian). German and Italian procedures and methods are illustrated in Figure 1 and Figure 2.

Distribution of participant group to study phases:

Medical geneticists are considered to be experts in precise genetic screening, advanced interpretation and results delivery to HCPs teams and patients. Medical geneticists involved in genetic screening will be recruited via purposive sampling within clinical partners of Screen4Care project and be interviewed as experts. The results of these interviews will not lead to any specific analyses but ensure the adequacy of the interview guide and questions.

The second group consists of "healthy" expecting parents from the general population (with pregnancy monitoring in regular care) recruited in the same geographic areas as the expecting parents currently seeking genetic consultation for RDs in one of the clinical centres. Focus group will be run by trained personal at the recruiting centre (trained midwifes, research nurses, depending on available resources). Uppsala University will support in the implementation and conduct of the group interviews. As a precautionary measure, if both partners volunteer to the study; the mother and father from the parental couple, will be included in separate groups to avoid any influence they may have on each other during the discussions. Recruitment of "healthy" expecting parents will be conducted via midwifery and gynaecology practices in Germany and in Italy.

The third group of participants will include expecting parents who seek genetic consultation but who are not necessarily carriers of genetic variants related to RDs themselves.. Recruitment of expecting parents seeking genetic consultation will be conducted by clinical genetic centres that are part of the Screen4Care project in both Germany and Italy. Parents will be identified as they visit the clinical centres.

For the DCE survey, "healthy" expecting parents from the general population and expecting parents already seeking genetic consultation will be recruited. "Healthy" parents

For peer review only

from general population will be recruited via SurveyEngine[4] in Italy, and via invitation shared from participating centres networks in Germany. Parents already seeking genetic consultation will be recruited via invitation from the participating clinical centres in Germany and Italy. The study aims to recruit approximately 2084 participants in total across the two participating countries (see Figure 3). For the qualitative phase, a maximum sample of 4 experts (two in Germany and two in Italy), 40 expecting parents (representing 20 couples in each country) for the group interviews will be recruited. The recruitment will be operated by the clinical centres that have access to "healthy" expecting parents (total of 84 for the first part). For the quantitative phase, the survey sample will be about 1000 participants in each country (500 "healthy" expecting parents from general population and 500 expecting parents seeking genetic consultation) (see figure 1 and 2).

A priori sample-size calculations represent a challenge in DCE experiments. Most published choice experiments have a sample size of 10 to more than 3000 respondents [35]. However, minimum sample size depends on several criteria, including the question format, the complexity of the choice task, the desired precision of the results, and the need to conduct subgroup analyses [32]. A method for computing sample size was proposed by de Bekker-Grob et al., 2015 [33], however, as the article points out there is no analytic solution or power calculation that can be used to determine the appropriate sample size for a DCE unless the researcher has enough information to inform the selection of priors. Additionally, for the "healthy" expecting parents from general population sample we plan to use a blocked design (asking people to only complete a randomly assigned subset of the choice tasks, thereby limiting the cognitive burden on each participant). Given what we know, recruiting 1000 respondents in each country should be sufficient to answer the proposed research questions and provide enough information to identify preferences in these groups and comparisons across these groups with acceptable precision.

Adapting Methods to Ethical specificities

As German, and Italian regulations differ in their understanding of the risk level of focus group in terms of privacy and discomfort for the participants (e.g. genetic related conversation that could lead to sharing opinion or experience connected with genetics in front of other participants might be an argument for ethical committees' refusal in Italy). Methods have been

[4] Survey Engine is a third-party recruitment company. https://surveyengine.com/

adapted for example by conducting individual interviews for parents seeking genetic screening (more sensitive sample) only by clinical geneticist (in Italy). Any informational material provided before signing the informed consent will point out the specific risks and expected benefits for participants to take an informed decision before entering the protocol.

Study Procedures

Information of participants and informed consent

Each participant will receive detailed written information on the nature, objectives, possible risks/ benefits of the research (for both the focus groups and quantitative preference study participation). Prior to the qualitative interviews, participants will be given time to consider their involvement and ask questions about the study to the researchers. They will be informed that they can stop their participation in the study at each point. Participants can only take part in the study if informed consent form is signed beforehand. The investigators will keep the original signed informed consent in accordance with current requirements. For online survey participation, the informed consent will be included in the online survey and digitally signed by the participants.

Individual interviews

Semi-structured interviews will be conducted with members of the IMI S4C consortium to test the interview guide and associated questions. These interviews will be conducted in English. The interview will take approximately one hour and will be conducted at a location convenient for the interviewee, via telephone, or via video conferencing platform.

Feedback from the pilot interviews will be evaluated and the interview guides will be adapted accordingly for each country. After making the necessary changes, the interview guide will be translated to German and Italian. Native language speaking trained interviewers from clinical centers will conduct the interviews using the interview guide. Collection, recording, and reporting of data will ensure the privacy, health, and welfare of research subjects during and after the study and in accordance with the country-specific regulations.

Group interviews

For peer review only

The group interviews will be used to provide more precise evidence for the most important characteristics of gNBS when parents decide about their participation in genetic screening programs.

At the start of the interview the objective of the S4C project and the interview procedures will be explained to the interviewee and the researcher will confirm that informed consent was given before starting the interview. The interviewee must sign the consent form before the start of the interview. Once informed consent is confirmed, the researcher will start the recording device. After asking the questions in the interview guide, participants will be asked to rank a list of potential attributes (emanating from the literature review) from most to least important according to their personal decision to take part in gNBS. These rankings will be discussed, and following the discussions participants will be allowed to change their ranking. The interviewer will ask the interviewees if they want to add something to the interview.

We plan to conduct a minimum of four group interviews per country (Germany, Italy) (see Figure 1, 2 and for overall description see Figure 3) and continue until data satuarion. There will be eight group interviews, in total. A group interview consists of 8-10 members. The group interview will take about two hours and will be conducted at a location and on date most convenient for all participants.

Figure 1: Study Flowchart in Germany GOES HERE

Figure 2: Study Flowchart in Italy GOES HERE

Figure 3: Protocol's procedures GOES HERE

The interviews will be recorded and transcribed verbatim. The interviews will be analysed iteratively using a grounded theory approach based on a mix of inductive and deductive coding. Analyses will be conducted in *Atlas.ti* software. The coding procedure will be handled by two independent coders from each country. The outputs will then be translated into English for further use by the research team. To assure a proper standardisation of the interviews across languages, the domestic researchers will be trained by Uppsala's University researchers and

For peer review only

will also be provided a detailed interview guide to help them through the process of every interview.

The information given by the interviewee will be treated as confidential and will be processed anonymously. The collected data will be stored securely and viewed only by authorised researchers of the project. The processed answers will be used for publication of reports or articles as part of the S4C research project. The audio recordings specifically will be destroyed after completion of the study and no files will be stored at the recruiting centers.

Discrete Choice Experiment (DCE)

A survey will be fully developed after the final selection of attributes and levels. The final selection of attributes (and levels) to be included in the DCE will be based on the (focus group) interviews with medical geneticists and PLWRD's advocates. First, attributes ranked highest during the qualitative studies will be taken forward for consideration. For attributes to be included in the final DCE they should be unambiguous, not overlapping and clearly defined. Additionally, they should be important to the anticipated target population while at the same time clinically relevant. Therefore, the final selection of the most important attributes will be based on attribute ranking from the focus groups in combination with the perspective of experts on practicalities of gNBS. After selecting the final attributes, appropriate levels that reflect current practice will inform the attributes list for the DCE.

The survey instrument, including the DCE, will be pre-tested using think aloud methods in a convenience sample of 3 to 5 "healthy" expecting parents from the general population in each country, using think aloud interviews to review the results from the interviews and to determine if any adjustments need to be made to improve clarity. This process will be conducted in collaboration with EURORDIS and patient organisation representatives' members of the PAB. They will ensure accurate use of vocabulary by assisting in drafting and reviewing the included definitions and explanatory texts as well as educational content to ensure accessibility and readability to lay audiences.

The initial DCE experimental design will be generated via NGene 1.0 (ChoiceMetrics, 2011) and hypothetical choice questions with three alternatives will be created (of which one is the 'no screening' alternative). These alternatives will be characterised by varying attribute levels. To ensure design efficiency, a pilot with 100 respondents in each country will be

For peer review only

conducted. All participants will provide informed consent before entering the task. Based on the data retrieved in the pilot test, a multinomial logit (MNL) model will be fitted. Beta estimates will be used to assign priors for the final experimental DCE design, which is a *d*-efficient (Bayesian) design [29]. To increase efficiency of the design, multiple blocks of choice tasks will be generated which will later be randomly distributed of respondents in such a way that each respondent will answer only one block of approximately 10-15 choice tasks.

Ethical and legal aspects

The S4C IMI Project (grant agreement No. 101007757) has been approved by the Medical Ethics Committee of References. Ethical approval for the first part of the protocol has been approved in Germany, from University of Erlangen (22-246-B) the Freiburg University Ethics committee (23-1005-S1-AV), and in Italy (University of Ferrara: CE: 357/2023/Oss/AOUFe, and Hospedale Bambino Gesu: No.2997 of 2 November 2023, Prot. No. _902). Ethical Review Authorities in Sweden have approved the protocol for data storage and handling at Uppsala University (2022-05806-01).

The ethical procedure will be performed at two distinct stages, the first for the qualitative part and the second for the quantitative part (DCE). As the first part will be used as an input and will inform the second one, ethical committee may not consider approving a "tentative" survey draft or a "tentative" list of attributes as it can significantly change the ethical risk for anonymity (if one attribute is "cost" compared to "pregnancy planning"). The overall S4C project also ensured an ethical framework with the Code of Ethics Practice that was developed internally as a project Deliverable and agreed with all WP leaders to inform each researcher within the S4C consortium about the main ethical risks with no exception of this specific task on preferences studies.

Confidentiality

Due to the interaction of the researchers with the interviewees it is impossible to collect the data anonymously by the recruiting centers, but the obtained information will be coded to allow confidential and anonymous processing and reporting of the data from the research team. The identity of the participants will not be revealed so the privacy will be guaranteed. The research data will be used for publications in scientific journals and writing of reports or

For peer review only

publication for the S4C project but will be registered and saved with a secure code. The coded anonymous data can be shared within the members of the consortium. The code, being the identification numbers linked to the identity of the participants, will be held confidential within the group of involved researchers. Only the researchers that are involved in this study will have access to this code and only they will know the identity of the participating stakeholder members.

Uppsala University will provide secure data storage space with restricted access to researcher analysing the data from the S4C partners involved in task 3.1. All digital data will be stored in Uppsala University's officially storage of research data (Dataportal Allvis) with specific ethical approval from The Swedish Ethical Review Authority, as Swedish Regulation requires. The data is physically stored in two redundant datacentres following (non-certified) ISO 27001 guidelines. Dataportal Allvis meets KRT value 233. Both datacentres are equipped with enterprise grade network, servers and hard disks with high availability, security and safety. All data centres are equipped with access controls, cooling systems, backup power and surge protection. They are protected against common threats such as fire, water damage, burglary, and theft. Access is restricted to technical staff and strictly protocolled. All data is encrypted in transit (https) and at rest (through database encryption). Allvis is configured with versioning of files, saving up to 500 versions of an individual file. A recycle bin saves deleted files up to 30 days. Content databases are configured with transactional logging, limiting potential data losses to 1 hour during critical failures. Databases are backed up incrementally, daily, weekly, monthly, and yearly using a TSM-based backup system hosted in the same data centers. Network traffic is protected through a robust firewall and servers hosting file content are not directly connected to the internet. The involved researchers commit to the highest standards of data security and protection to preserve the personal rights and interests of study participants. Participants will be informed that they can contact Uppsala University data security services for any information via email (dataskyddsombud@uu.se).

The involved researchers commit to the highest standards of data security and protection to preserve the personal rights and interests of study participants. They will adhere to the provisions set out in:

- The General Conference of the United Nations Educational, Scientific and Cultural Organization (UNESCO), meeting in Paris, from 9 to 24 November 2021, at its 41st session for Open science recommendations.

- A central guide for all research that includes human subjects will be the Declaration of Helsinki - Ethical principles for medical research involving human subjects from the World Medical Association (WMA Adopted by the 18th WMA General Assembly, Helsinki, Finland, June 1964 -amended by the 64th WMA General Assembly, Fortaleza, Brazil, October 2013
- Directive 2002/58/EC of the European Parliament and of the Council of 12 July 2002 concerning the processing of personal data and the protection of privacy in the electronic communications sector (Directive on privacy and electronic communications)
- Regulation (EU) 2016/679 of the European Parliament and of the Council of 27 April 2016 on the protection of natural persons regarding the processing of personal data and on the free movement of such data, and repealing Directive 95/46/EC (General Data Protection Regulation) (Text with EEA relevance)
- Regulation (EU) 2018/1725 of the European Parliament and of the Council of 23 October 2018 on the protection of natural persons regarding the processing of personal data by the Union institutions, bodies, offices, and agencies and on the free movement of such data, and repealing Regulation (EC) No 45/2001 and Decision No 1247/2002/EC

Data analysis

Using the systematic literature review, a list of tentative attributes will be extracted from the thematic analysis based on gNBS themes and recurrent factors. The list will also include psychological factors (e.g. depression, anxiety, emotion regulation) that will have to be addressed as contextual elements separately. As the literature extensively points at the psychosocial impact of gNBS, even in 2023 (Blom et al., 2023; Tobik et al., 2023), we decided to include the main psychological measures to ensure possibility for subgroup analysis based on validated measures to better describe the sample but also be able to potentially describe differences in preferences within and between the populations.

As numerous potential attributes are expected to be highlighted during the literature review, the focus group will help clarify the most important elements. Independent coders will analyse the transcription of the focus group and provide content analysis. In order to refine the attribute list, the ranking task will be performed presenting 10-15 attributes to design the DCE. The data from the ranking task will be analyzed to understand what attributes are most

For peer review only - http://bmjopen.bmj.com/site/about/guidelines.xhtml

important to expecting parents. This information will be considered when deciding the final attributes for the DCE.

Participant characteristics that will be reported to describe the study samples include demographic characteristics (age, gender, pregnancy stage, income level among others), but also attitudes and psychosocial factors such as health literacy, and anxiety for example (Hospital Anxiety and Depression Scale, available in both Italian and German; Annunziata et al., 2011; Hinz & Brähler, 2011). In collaboration with recruiting centers, we will consider the inclusion of personality scales, emotion regulation scales and-or coping strategies scales to provide a more precise psychological assessment.

Preferences and understanding of the survey instrument (comprehension questions) will be assessed by examining the relationship between psychological instruments of cognitive ability (e.g. health literacy and numeracy) and survey responses. This includes comparing responses to warm up questions, survey internal validity checks, and attitudinal questions about reported complexity of tasks across various levels of each psychological instrument.

To determine preferences of study participants regarding gNBS, attributes, level estimates and the conditional relative importance of attributes will be reported in the DCE using random parameters logit (RPL) modelling and latent class analyses (LCA). Heterogeneity of preferences and the impact of participant characteristics (e.g. demographics, psychological validated scales) will be investigated by applying appropriate statistical models including LCA for the DCE and/or subgroup analyses (RPL). All results described above will be compared between the German and Italian study samples to determine whether there are cross-country differences. The results of the quantitative part of the preference study is planned to be published in scientific journal (Open Access) and to be shared in all S4C project communication networks to reach out to different audiences as part of the general communication plan of the project.

For peer review only

Acknowledgements:

Author contributions: Sylvia Martin (SM), Joshua Coulter (JC), Jorien Veldwijk (JV) drafted the protocol and closely ensures the adaptation of the final version to all authors's comments.

SM, JC, JV, Åsa Grauman (AG), Brett Hauber (BH), Mats Hansson (MH), contributed to the design and implementation of the protocol.

Planning of the work, conduct and reporting was ensure by all authors and included clinicians for the process of ethical application and procedure, but also to design the protocol in adequacy with clinical requirements. Ethical procedure was led by SM, MA, Angolini Emanuele (AE), Enrico Silvio Bertini (ESB), Lucia Pia Bruno (JPB), Alessandra Ferlini (AF), Fernanda Fortunato (FF), Vera Frankova (VF), Janbernd Kirschner (JK), Ferdinand Knieling (FK), Silvia Ottombrino (SO), Antonio Novelli (AN), Roman Raming (RR), Christina Saier (CS), Stefaan Sansen (SS) and Nicolas Garnier (NG). Edith Gross (EG) coordinated the patients and patient's advocacy participation for both ethical procedure and protocol drafting.

SM, Gergana Kyosovksa (GK), Jennifer Audi (JA), drafted the first version of the project and included all relevant ethical consideration and introduction information.

FK, RR, JK, SS, CS, LPB, VF, EG, JA, GK, FF, AF, NG, ESB, AE, SO, AN, SM, JC, JV, AG, BH, MH, drafted the precise clinical information used in the draft, checked for relevance of the vocabulary and reviewed the overall structure of the article.

Non-authors contribution: We thank the members of the Screen 4 Care Patient advisory board for their constructive help during consultation meeting and for the quality of their written feedback.

Funding statement: 'This work was supported by Sreen4Care. This project has received funding from the Innovative Medicines Initiative 2 Joint Undertaking (JU) under grant agreement **No 101034427**. The JU receives support from the European Union's Horizon 2020 research and innovation programme and EFPIA.

Competing interests' statement: No competing interest.

Data statement: Data will be made available under reasonable demand to the corresponding authors and in accordance to Screen 4 Care data security procedures.

For peer review only - http://bmjopen.bmj.com/site/about/guidelines.xhtml

References:

[1] Nguengang Wakap, S., Lambert, D. M., Olry, A., Rodwell, C., Gueydan, C., Lanneau, V., Murphy, D., Le Cam, Y., & Rath, A. (2020). Estimating cumulative point prevalence of rare diseases: analysis of the Orphanet database. *European journal of human genetics: EJHG*, *28*(2), 165–173. https://doi.org/10.1038/s41431-019-0508-0

[2] Hay E, Elmslie F, Lanyon P and Cole T. The Diagnostic Odyssey in rare diseases; a Task and Finish Group report for the Department of Health and Social Care [version 1; not peer reviewed]. *NIHR Open Res* 2022, **2**:3 (document) (https://doi.org/10.3310/nihropenres.1115171.1)

[3] Marwaha, S., Knowles, J. W., & Ashley, E. A. (2022). A guide for the diagnosis of rare and undiagnosed disease: beyond the exome. *Genome medicine*, *14*(1), 1-22.

[4]Ferreira C. R. (2019). The burden of rare diseases. *American journal of medical genetics. Part A*, *179*(6), 885–892. https://doi.org/10.1002/ajmg.a.61124

[5] Gokdemir, Y., Eyuboglu, T. S., Emiralioglu, N., Er, B., Sen, V., Pekcan, S., ... & Yılmaz, O. (2021). Geographical barriers to timely diagnosis of cystic fibrosis and anxiety level of parents during newborn screening in Turkey. *Pediatric Pulmonology*, *56*(10), 3223-3231.

[6] Grob, R., Roberts, S., & Timmermans, S. (2018). Families' Experiences with Newborn Screening: A Critical Source of Evidence. *Hastings Center Report*, *48*, S29-S31.

[7] Bailey Jr, D. B., Bishop, E., Raspa, M., & Skinner, D. (2012). Caregiver opinions about fragile X population screening. *Genetics in Medicine*, *14*(1), 115-121.

[8] van Dijk, T., Kater, A., Jansen, M., Dondorp, W. J., Blom, M., Kemp, S., ... & Henneman, L. (2021). Expanding Neonatal Bloodspot Screening: A Multi-Stakeholder Perspective. *Frontiers in pediatrics*, *9*, 706394.

[9] Chavez-Yenter, D., Vagher, J., Clayton, M. F., Rindler, M., Shukovich, M., & Kaphingst, K. A. (2021). "Being proactive, not reactive": exploring perceptions of genetic testing among White, Latinx, and Pacific Islander Populations. *Journal of Community Genetics*, *12*(4), 617-630.

[10] Perobelli, S., Zanolla, L., Tamanini, A., Rizzotti, P., Maurice Assael, B., & Castellani, C. (2009). Inconclusive Cystic Fibrosis neonatal screening results: long-term psychosocial effects on parents. *Acta Paediatrica*, *98*(12), 1927-1934.

[11] Pruniski, B., Lisi, E., & Ali, N. (2018). Newborn screening for Pompe disease: Impact on families. *Journal of inherited metabolic disease*, *41*(6), 1189-1203.

[12] Bailey Jr, D. B., Berry-Kravis, E., Gane, L. W., Guarda, S., Hagerman, R., Powell, C. M., ... & Wheeler, A. (2017). Fragile X newborn screening: lessons learned from a multisite screening study. *Pediatrics*, *139*(Supplement_3), S216-S225.

[13] Friedman, J.M., Cornel, M.C., Goldenberg, A.J. *et al.* Genomic newborn screening: public health policy considerations and recommendations. *BMC Med Genomics* **10**, 9 (2017). https://doi.org/10.1186/s12920-017-0247-4

[14] Knoppers, B. M., Sénécal, K., Borry, P., & Avard, D. (2014). Whole-genome sequencing in newborn screening programs. *Science translational medicine*, *6*(229), 229cm2-229cm2. DOI: 10.1126/scitranslmed.3008494

For peer review only - http://bmjopen.bmj.com/site/about/guidelines.xhtml

[15] Ozdemir, S., Finkelstein, E., Lee, J. J., Too, I. H. K., Teo, K. Y. C., Tan, A. C. S., ... & Cheung, G. C. M. (2022). Understanding patient preferences in anti-VEGF treatment options for age-related macular degeneration. *PloS one*, *17*(8), e0272301.

[16] Moultrie, R. R., Paquin, R., Rini, C., Roche, M. I., Berg, J. S., Powell, C. M., & Lewis, M. A. (2020). Parental views on newborn next generation sequencing: implications for decision support. *Maternal and child health journal*, *24*, 856-864.

[17] Bombard, Y., Miller, F. A., Hayeems, R. Z., Barg, C., Cressman, C., Carroll, J. C., ... & Chakraborty, P. (2014). Public views on participating in newborn screening using genome sequencing. *European Journal of Human Genetics*, *22*(11), 1248-1254.

[18] Plass, A. M. C., van El, C. G., Pieters, T., & Cornel, M. C. (2010). Neonatal screening for treatable and untreatable disorders: prospective parents' opinions. *Pediatrics*, *125*(1), e99-e106.

[19] Bailey, D. B., Lewis, M. A., Harris, S. L., Grant, T., Bann, C., Bishop, E., ... & Therrell, B. L. (2013). Design and evaluation of a decision aid for inviting parents to participate in a fragile X newborn screening pilot study. *Journal of genetic counseling*, *22*(1), 108-117.

[20] Miller, F. A., Hayeems, R. Z., Bombard, Y., Cressman, C., Barg, C. J., Carroll, J. C., ... & Regier, D. A. (2015). Public perceptions of the benefits and risks of newborn screening. Pediatrics, 136(2), e413-e423.

[21] Wright, S. J., Ulph, F., Lavender, T., Dharni, N., & Payne, K. (2018). Understanding midwives' preferences for providing information about newborn bloodspot screening. *MDM policy & practice*, *3*(1), 2381468317746170.

[22] Loeber, J. G., Platis, D., Zetterström, R. H., Almashanu, S., Boemer, F., Bonham, J. R., Borde, P., et al. (2021). Neonatal Screening in Europe Revisited: An ISNS Perspective on the Current State and Developments Since 2010. *International Journal of Neonatal Screening*, *7*(1), 15. MDPI AG. Retrieved from http://dx.doi.org/10.3390/ijns7010015

[23] Hammond, J., Klapwijk, J. E., Riedijk, S., Lou, S., Ormond, K. E., Vogel, I., ... & Lewis, C. (2022). Assessing women's preferences towards tests that may reveal uncertain results from prenatal genomic testing: Development of attributes for a discrete choice experiment, using a mixed-methods design. *PloS one*, *17*(1), e0261898.

[24] Buchanan, J., Hill, M., Vass, C. M., Hammond, J., Riedijk, S., Klapwijk, J. E., ... & Lewis, C. (2022). Factors that impact on women's decision-making around prenatal genomic tests: An international discrete choice survey. *Prenatal diagnosis*, *42*(7), 934-946.

[25] Hensher, D., J.M. Rose, and W.H. Greene, *Applied Choice Analysis: second edition*. 2015, Cambridge, UK: Cambridge University Press.'

[26] Hiligsmann, M., et al., Nominal group technique to select attributes for discrete choice experiments: an example for drug treatment choice in osteoporosis. Patient Prefer Adherence, 2013; 7:133-9

[27] Reed Johnson, F., E. Lancsar, D. Marshall, V. Kilambi, A. Muhlbacher, D.A. Regier, B.W. Bresnahan, B. Kanninen, and J.F. Bridges, Constructing experimental designs for discrete-choice experiments: report of the ISPOR Conjoint Analysis Experimental Design Good Research Practices Task Force*.* *Value Health*, 2013. 16(1): p. 3-13.

[28] Bridges, J.F., A.B. Hauber, D. Marshall, A. Lloyd, L.A. Prosser, D.A. Regier, F.R. Johnson, and J. Mauskopf, Conjoint analysis applications in health--a checklist: a report of the

For peer review only - http://bmjopen.bmj.com/site/about/guidelines.xhtml

ISPOR Good Research Practices for Conjoint Analysis Task Force. *Value Health*, 2011. 14(4): p. 403-13.

[29] Kessels, R., et al., An efficient algorithm for constructing bayesian optimal choice designs. Journal of Business & Economic Statistics, 2009; 27(2):279-291.

[30] Louviere, J.J., et al., Designing discrete choice experiments: do optimal designs come at a price? Journal of Consumer Research, 2008; 35(2):360-375.

[31] Marshall, D., et al., Conjoint Analysis Applications in Health - How are Studies being Designed and Reported?: An Update on Current Practice in the Published Literature between 2005 and 2008. Patient., 2010; 3(4):249-56.

[32] Louviere, J., D. Hensher, and J. Swait, Stated Choice Methods; analysis and application. 2000, Cambridge: Cambridge University Press.

[33]de Bekker-Grob, E.W., B. Donkers, M.F. Jonker, and E.A. Stolk, Sample Size Requirements for Discrete-Choice Experiments in Healthcare: a Practical Guide. *Patient*, 2015. 8(5): p. 373-84.

[34] Rose, J. M., & Bliemer, M. J. C. (2009). Constructing efficient stated choice experimental desings. *Transport Reviews, 9, 1–31*.

[35] Merlo, G., van Driel, M., & Hall, L. (2020). Systematic review and validity assessment of methods used in discrete choice experiments of primary healthcare professionals. *Health economics review*, *10*(1), 1-9.

For peer review only

Figure Legend:

Figure 1- Study Flowchart in Germany

Figure 2- Study Flowchart in Italy

Figure 3- Protocol's procedures

For peer review only

For peer review only - http://bmjopen.bmj.com/site/about/guidelines.xhtml

For peer review only

For peer review only - http://bmjopen.bmj.com/site/about/guidelines.xhtml

Figure 1: Study Flowchart in Germany

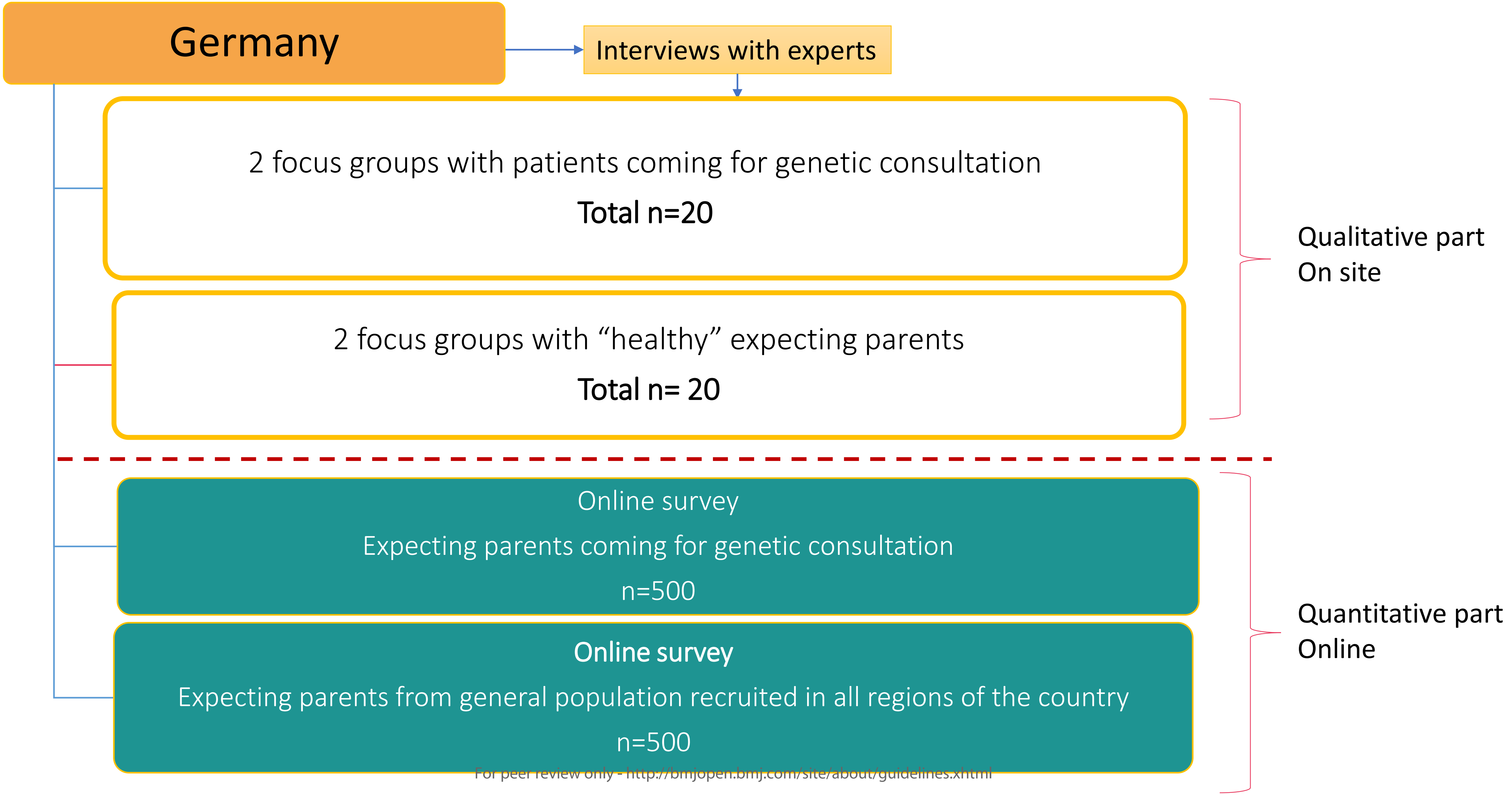

For peer review only - http://bmjopen.bmj.com/site/about/guidelines.xhtml

Figure 2: Study Flowchart in Italy

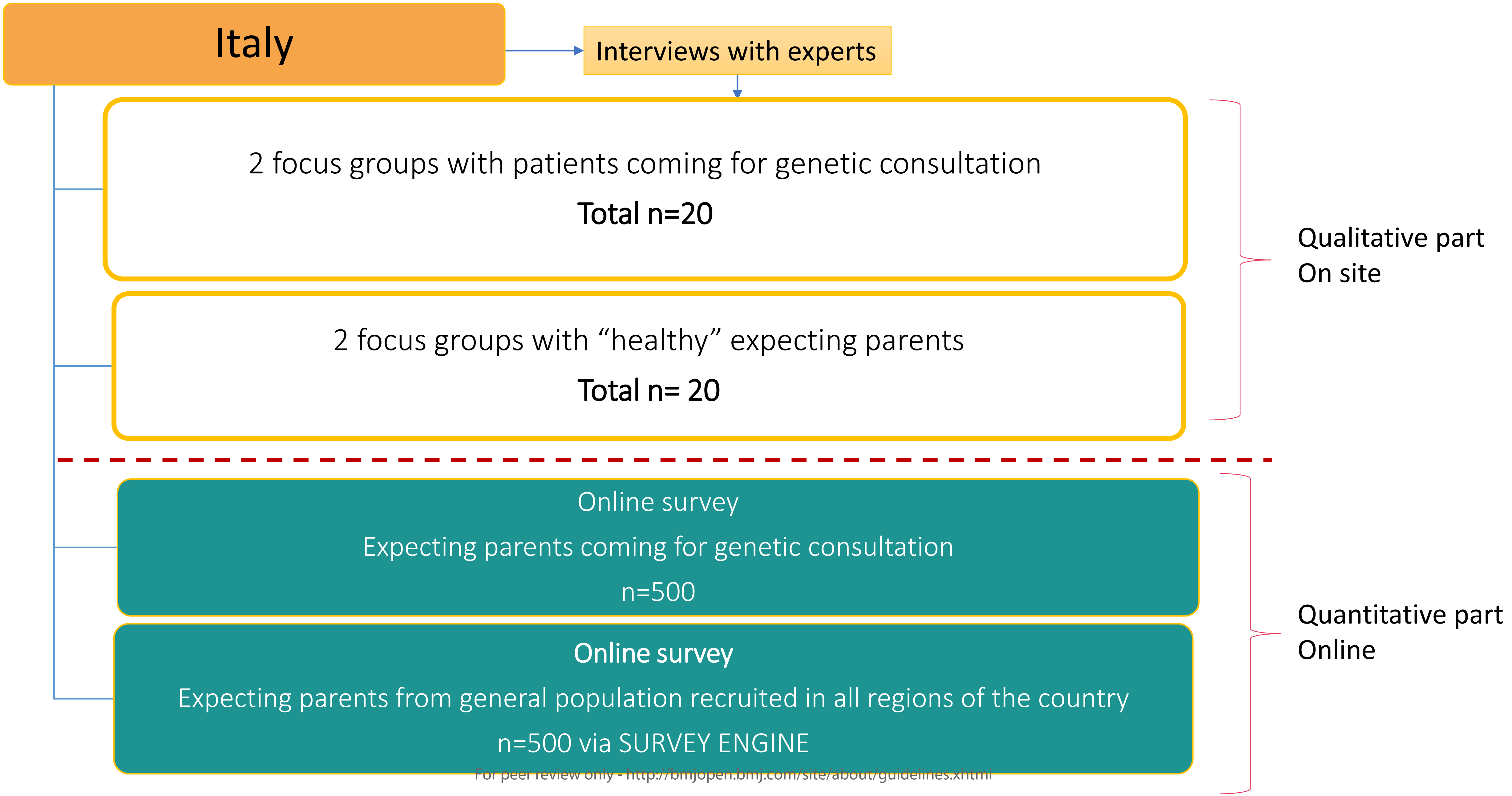

Figure 3: Protocol's procedures

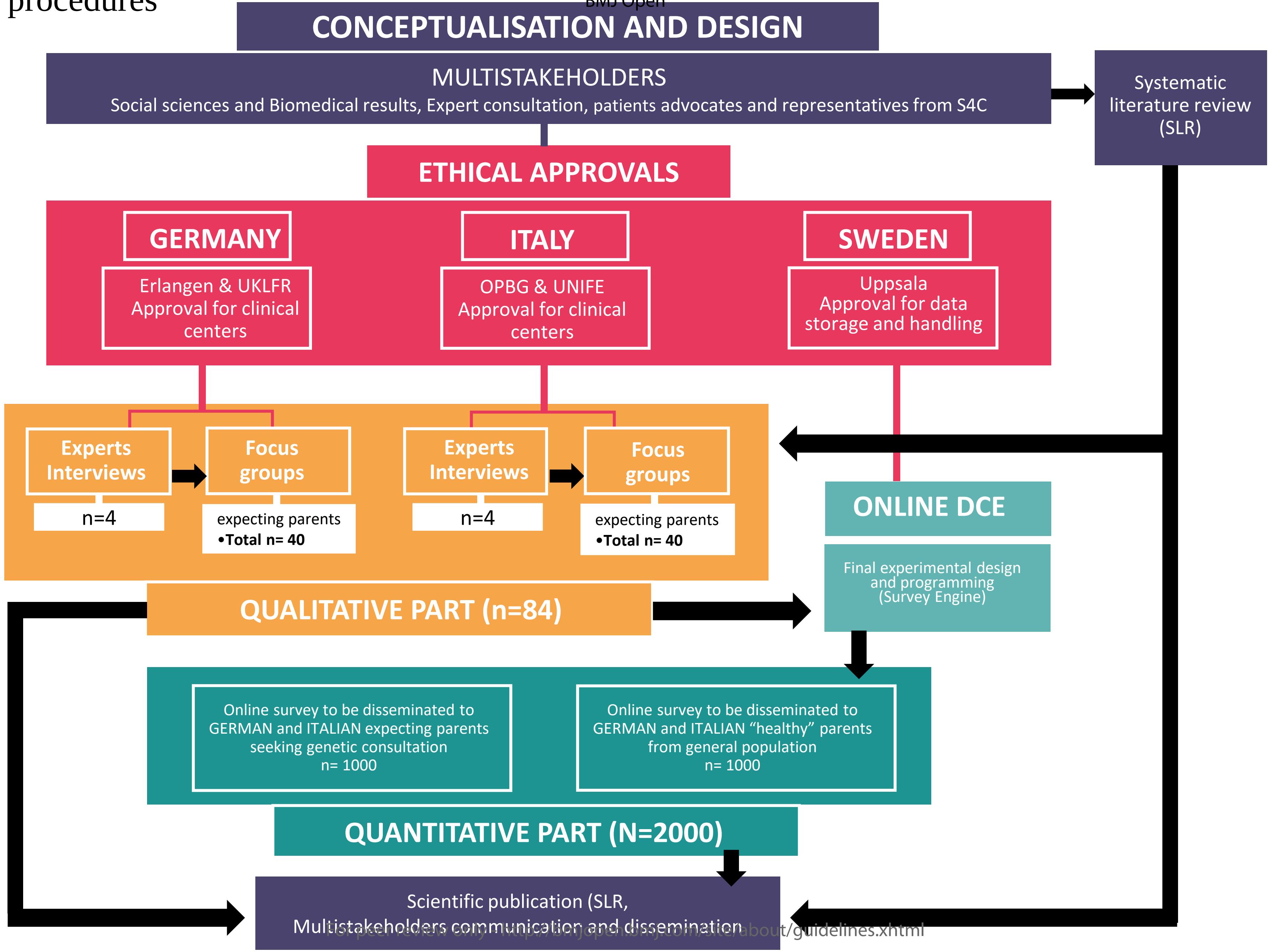

BMJ Open

Patient Preferences in Genetic Newborn Screening for Rare diseases: study protocol

Journal:	*BMJ Open*
Manuscript ID	bmjopen-2023-081835.R2
Article Type:	Protocol
Date Submitted by the Author:	05-Mar-2024
Complete List of Authors:	MARTIN, Sylvia; Uppsala Universitet, Center for Research and Bioethics<br>Angolini, Emanuele; Ospedale Pediatrico Bambino Gesù, Research Unit of Neuromuscular and Neurodegenerative Disease<br>Audi, Jennifer; Takeda Pharmaceuticals International AG<br>Bertini, Dr. Enrico; Ospedale Pediatrico Bambino Gesù, Research Unit of Neuromuscular and Neurodegenerative Disease<br>Bruno, Lucia Pia; University of Siena, Medical Genetics; Telethon Institute of Genetics and Medicine<br>Coulter, Joshua; Pfizer Global Pharmaceuticals<br>Ferlini, Alessandra; University of Ferrara, Department of Medical Sciences<br>Fortunato, Fernanda; University of Ferrara, Department of Medical Sciences<br>Frankova, Vera; Charles University First Faculty of Medicine<br>Garnier, Nicolas; Pfizer Inc<br>Grauman, Åsa; Uppsala Universitet, Center for Research and Bioethics<br>Gross, Edith; EURORDIS<br>Hauber, Brett; Pfizer Inc<br>Hansson, Mats; Uppsala Universitet, Center for Research and Bioethics<br>Kirschner, Janbernd; University of Freiburg<br>Knieling, Ferdinand; Erlangen University Hospital<br>Kyosovksa, Gergana; Bulgarian Association for Personalised Medicine<br>Ottombrino, Silvia; Ospedale Pediatrico Bambino Gesù, Research Unit of Neuromuscular and Neurodegenerative Disease<br>Novelli, Antonio; Ospedale Pediatrico Bambino Gesù, Research Unit of Neuromuscular and Neurodegenerative Disease<br>Raming, Roman; Erlangen University Hospital<br>Sansen, Stefaan; Sanofi-Aventis SA, Department of Medical Biotechnologies<br>Saier, Christina; Faculty of Medicine, Department of Neuropediatrics and Muscle Disorders<br>Veldwijk, Jorien; Erasmus Universiteit Rotterdam
**Primary Subject Heading**:	Genetics and genomics
Secondary Subject Heading:	Qualitative research, Research methods
Keywords:	GENETICS, QUALITATIVE RESEARCH, Patient-Centered Care, Patient Participation, Patient Reported Outcome Measures

SCHOLARONE™
Manuscripts

Patient Preferences in Genetic Newborn Screening for Rare diseases: study protocol

Authors: Sylvia Martin[1*], Angolini Emanuele[14], Jennifer Audi[5], Enrico Silvio Bertini[14], Lucia Pia Bruno[7,8], Joshua Coulter[3], Alessandra Ferlini[12], Fernanda Fortunato[12], Vera Frankova[9], Nicolas Garnier[3], Åsa Grauman[1], Edith Gross[13], Brett Hauber[3], Mats Hansson[1.], Janbernd Kirschner[10], Ferdinand Knieling[6], Gergana Kyosovksa[4], Silvia Ottombrino[14], Antonio Novelli[14], Roman Raming[6], Stefaan Sansen[8], Christina Saier[10], Jorien Veldwijk [1,2].

Word count: 5933

Funding: Sreen4Care project, Innovative Medicines Initiative grant agreement No 101034427.

1. Centre for Research and Bioethics (CRB), Uppsala University, Sweden, Husargatan 3, BMC, entrance A11.

2. Erasmus School of Health Policy & Management, Erasmus University Rotterdam, P.O.Box 1738, 3000 DR Rotterdam, The Netherlands.

3. Pfizer, 235 E 42nd St, New York, USA.

4. Bulgarian Association for Personalised Medicine, 45 Bacho Kiro str., 1202 Sofia, Bulgaria.

5. Takeda Pharmaceuticals International AG, Zurich, Switzerland

6. Departmenf of Pediatrics and Adolescent Medicine, Universitätsklinikum Erlangen, Friedrich-Alexander-Universität (FAU) Erlangen-Nürnberg, Erlangen, Germany.

7. Medical Genetics, University of Siena, Siena, Italy

8. Med Biotech Hub and Competence Center, Department of Medical Biotechnologies, University of Siena, Siena, Italy.

9. Institute for Medical Humanities, Charles University First Faculty of Medicine, Karlovo náměstí 40, 128 00 Prague 2, Czech Republic

For peer review only

10. Department of Neuropediatrics and Muscle Disorders, Medical Center – University of Freiburg, Faculty of Medicine, Freiburg, Germany

11. Sanofi, Diegem, Belgium.

12. Unit of Medical Genetics, Department of Medical Sciences, University of Ferrara, Via Fossato di Mortara, 74, 40122 Ferrara (Italy)

13. EURORDIS, Plateforme Maladies Rares,96, rue Didot, 75014 Paris (France).

14. Research Unit of Neuromuscular and Neurodegenerative Disease, Ospedale Pediatrico Bambino Gesu, Roma, Lazio (Italy).

* Sylvia Martin is the corresponding author: Centre for Research and Bioethics (CRB), Uppsala University, Sweden, Husargatan 3, BMC, entrance A11. sylvia.martin@crb.uu.se

For peer review only

Abstract:

Introduction: Rare diseases collectively impact over 30 million people in Europe. Most individual conditions have a low prevalence which has resulted in a lack of research and expertise in this field, especially regarding genetic newborn screening (gNBS). There is increasing recognition of the importance of incorporating patients' needs and general public perspectives into the shared-decision making process regarding gNBS. This study is part of the Innovative Medicine Initiative project Screen 4 Care which aims at shortening the diagnostic journey for rare diseases by accelerating diagnosis for patient living with rare diseases through genetic newborn screening (gNBS) and the use of digital technologies, such as Artificial Intelligence (AI) and Machine Learning (ML). Assessing expecting parent's perspectives, attitudes and preferences regarding gNBS for rare diseases in Italy and Germany.

Methods and analysis: A mixed method approach will assess perspectives, attitudes and preferences of (1) expecting parents seeking genetic consultation and (2) "healthy" expecting parents from general population in two countries (Germany and Italy). Focus groups and interviews using the nominal group technique and ranking exercises will be performed (qualitative phase). The results will inform the treatment of attributes to be assessed via a survey and a discrete choice experiment (DCE). The total recruitment sample will be 2084 participants (approximatively 1000 participants in each country for the online survey). A combination of thematic qualitative and logit-based quantitative approaches will be used to analyze the results of the study.

Ethics and dissemination: This study has been approved by the Erlangen University Ethics Committee (22-246_1-B), the Freiburg University Ethics committee (23-1005-S1-AV) and for clinical centers in Italy (University of FerraraCE: 357/2023/Oss/AOUFe, and Hospedale Bambino Gesu: No.2997 of 2 November 2023, Prot. No. _902), and approved for data storage and handling at Uppsala University (2022-05806-01). The dissemination of the results will be ensured via scientific journal publication (Open Access).

Strengths and limitations of this study:

- This study has been developed by an international expert team together with industry and patient representatives to provide an example of a rigorously designed 2-stepped preferences study.

For peer review only

-This study will collect data from a significantly large sample across several populations, allowing comparison of gNBS uptake perspectives, attitudes and preferences, psychological variables, and socio-demographic information in two European countries.

- Only the most important characteristics of gNBS can be incorporated into the DCE, therefore considerations of less important characteristics can only be interpreted based on the outcomes of the qualitative work and have to be assumed less important (on average) than the least important outcome of the DCE.

-Study elements might be complex (cross cultural elements, potential issues in translations, recruitment populations differences) and prone to misunderstanding even if high involvement of patient representatives in the development of the study material will reduce these risks.

-This study will recruit participants from different countries where the in-place gNBS procedure is differently implemented in the general care settings.

Keywords: genetic newborn screening, rare disease, patient preference, discrete choice experiment.

For peer review only

Introduction

There are more than 7,000 known rare diseases (RDs), conditions that affect one, or less than one, individual in 2,000 (point prevalence of <1/1 000 000 in more than 84% of RDs [1]). People living with rare a disease (PLWRD[1]) typically face an arduous journey to proper diagnosis, enduring on average eight years of countless physician consultations [2]. Time to diagnosis is a stressful period during which patients are faced with inconclusive test outcomes, possible misdiagnoses, and thereby ineffective treatments and healthcare resource utilisation [3].

Although RDs collectively impact over 30 million people in Europe, most individual conditions have a low prevalence [4]. This has resulted in a lack of research and expertise in this field. Difficulties in the process of receiving an accurate diagnosis and uncertainty around experienced symptoms continue to add to the difficulties that PLWRD experience, but also affecting their families, caregivers, physicians, and society as a whole [5,6]. Currently, there is increased recognition of the importance of incorporating patients' needs and perspectives into the shared decision-making process and providing more avenues for engagement and involvement of PLWRD at each stage of the design and development of health interventions. Consequently, PLWRDs play a vital role in the implementation of novel solutions that are designed to improve their own life and health.

The rapid evolution of genetic diagnostic techniques, including gene panels, whole exome, and whole genome sequencing, will provide new opportunities for early diagnosis of RDs potentially enabling PLWRDs to also have better access to therapies and treatments. Thus, genetic newborn screening (gNBS)[2] could lead to a public health paradigm shift with early diagnosis and intervention which may prevent health damage before it is irreversible and avoid functional limitations leading to premature death in potentially preventable diseases.

---

[1] This acronym has been selected based on its usage in international literature https://www.rarediseasesinternational.org/wp-content/uploads/2022/01/Final-UN-Text-UN-Resolution-on-Persons-Living-with-a-Rare-Disease-and-their-Families.pdf

[2] We will use a broad definition of genetic newborn screening as defined by Institute NHGR. 2023, 17th November [Available from: https://www.genome.gov/genetics-glossary/Newborn-Genetic-Screening : "*Newborn screening is a set of laboratory tests performed on newborn babies to detect a set of known genetic diseases. Typically, this testing is performed on a blood sample obtained from a heel prick when the baby is two or three days old. […].*"

For peer review only

Implementation of gNBS for RDs is thought to improve early access to care, redirect/focus treatment [7,8], impact pregnancy and family planning [9], and increase detection rate and accuracy of newborn screening programs as a whole [10].

However, undergoing gNBS has obvious ethical and social implications both for newborns and for parents as it may induce stress and increase concerns regarding diagnosis, treatment, and the child´s future [11,12]. Ethical and psycho-social issues related to gNBS present considerable dilemmas regarding the effects of genetic data on both the infant and their family, as well as with regard to broader societal aspects. These concerns play a critical role in shaping regulatory structures and health policies aimed at addressing these ethical obstacles (Reinsteing, 2015; Grob, 2019). The National Human Genome Research Institute *Newborn Screening Fact Sheet* states, *"with the decreasing cost of genome sequencing, there is potential for its clinical application in newborn screening. This could supplement or replace traditional panels of tests, providing more comprehensive health information. However, several questions remain about the practicality, ethics, and long-term implications of incorporating genome sequencing into routine newborn screening*" (https://www.genome.gov/about-genomics/fact-sheets/Newborn-Screening-Fact-Sheet, consulted on January 2 2024). Therefore, clarifying HCPs', patients', and general public perspectives, attitudes and preferences on gNBS implications may improve its safe and equitable development.

Additionally, there are challenges related to interpretation of gNBS results, especially if based on whole-exome or whole-genome sequencing approaches: communication of test results, management of incidental findings and/or of variants of uncertain significance, data storage, and other issues that need to be addressed [13,14].

Bearing in mind that gNBS and its results may impact society and the individual itself, their adoption and implementation in medical practice depends on attitudes, experiences, and preferences of different stakeholders. This study aims to understand the general public's perspectives and attitudes of gNBS and their preferences towards gNBS. Of particular interest are any differences in perspectives, attitudes and preferences between medical geneticists, expecting parents seeking genetic consultation during their pregnancy, and 'healthy' expecting parents.

There has been an increase in preference studies on genetic screening. These studies examine various aspects and challenges from both patients and health care professional stakeholders [15]. Even if the general public's attitude towards gNBS for RDs tends to be

For peer review only

positive [16-18], research also demonstrated that the acceptance of gNBS in the general public does not reflect the uptake of gNBS during pregnancy [19].

The first part of our protocol describes a systematic literature review of preference study results for gNBS (PROSPERO: CRD42022297678) in order to inform the design of the qualitative and quantitative parts of the protocol. Specific methods like discrete choice experiment (DCE) for eliciting preferences for gNBS have not been thoroughly implemented in Europe and only two previous DCEs studies in this field were identified. Miller et al. (2015) [20] assessed the perception of lay audiences regarding gNBS in Canada. Respondents from the general public were positive about the potential for gNBS expansion in the country based on the expected clinical benefits, improvement of reproductive risk management, and also the possibility of earlier diagnosis. Wright et al., (2018) [21], in another DCE, investigated information provision among midwives in the UK; when asking them about when to disclose information to expecting parents, or what type of information should be provided. According to this study, the potential for receiving a "false positive" result should not be disclosed (as it may not be helping decision making), and the best period to provide information would be late pregnancy to 3 days post-birth. The current study protocol, 7 years after the first DCEs on the topic in Europe [20], offers a comparison point for diverse cultural perspectives, attribute importance and technical advances in genetic techniques preferences. Moreover, this study will allow us to assess the perspective of gNBS in Europe among expecting parents who might face such a decision as health care systems are on the verge of implementing or extending gNBS programs. In fact, most of the European countries that implemented neonatal screening in the 60s/ 70s are now implementing an extended panel, envisioning the potential to screen for 40 to 50 conditions to be tested with a single blood spot using improvements in molecular technologies [22]. Protocols for gNBS research offer innovations for prompt and efficient treatment, as well as reducing obstacles related to worry in parents or over diagnosis (Gray et al., 2008). The research about gNBS serves as a catalyst for ongoing advancements in screening's sensitivity and specificity. The goal of the screening process optimization efforts is to provide assurance that true positive cases will be accurately diagnosed and to prevent families from being upset by an unexpected false positive result or from experiencing severe anxiety as an effect of results with uncertain significance (La Marca et al., 2023). This study will inform both clinical research and patient advocates stakeholders contributing to gNBS for RDs field.

For peer review only

A recent international research protocol (6 countries) addressed the preferences of women toward prenatal screening results [23], with the same stepwise structure as our protocol but in a connected field (prenatal testing). Based on a systematic literature review, they extracted 19 tentative attributes and refined them into 12. On the same prenatal topic, Buchanan et al. in 2022 [24] evaluated womens' decision making on prenatal genomic screening with a DCE (12 scenarios). These results were collected on an international (8 countries) level including a wide range of cultural examples from China to the United States, and showed that women looked for high diagnostic yield, short turnaround times and uncertain results to be reported (both variant of unknown significance and secondary findings).

These attributes and choices may be considered for neonatal gNBS but they may be different as the pregnancy planning decisions are not at stake anymore and the attributes of importance for new parents may be different after birth of their infant. Our research will assess the perspectives, attitudes and preferences from a more targeted population of expecting parents.

Methods and analysis

Research Aims and Objectives: This study is part of the larger project entitled 'Screen4Care (S4C)'. S4C is a research project launched in October 2021, which aims at shortening the diagnostic journey for rare diseases by accelerating diagnosis for PLWRDs through gNBS and digital technologies such as Artificial Intelligence (AI) and Machine Learning (ML). S4C has received funding from the Innovative Medicines Initiative 2 (IMI2) Joint Undertaking (JU). IMI, now superseded by IHI (Innovative Health Initiatives), is the world's largest public-private partnership in the life sciences whose mission is to "*improve health by speeding up the development of, and patient access to, innovative medicines, particularly in areas where there is an unmet medical or social need*."

The expected start of the study is December 2023 for a planned end in July 2024. The study has several objectives, being:

- To measure perspectives and attitudes of medical geneticists, parents seeking genetic consultation, and "healthy" expecting parents for gNBS.
- To elicit preferences of "healthy" expecting parents from the general population as well as of parents seeking genetic consultation.
- To identify and understand any heterogeneity in preferences for gNBS based on demographic characteristics and attitudes towards screening and genetic testing in general.

For peer review only

- To provide predicted uptake rates of gNBS based on the willingness to take part in rare disease screening program based on predefined characteristics of the screening test and output (not disease-specific).

Patient and Public involvement: Research questions and information materials have been developed with patient representatives.

Design of the study

A discrete choice experiment technique (DCE) will be used in this study, which is a globally established technique to elicit preferences in areas such as marketing, environmental economics, transportation, and health economics [25-28]. DCEs are based on Random Utility Theory [29-31]. Respondents are asked to complete several 'choice-tasks' (online), each of which consists of two or more alternatives that describe a treatment at hand. This description is based on treatment characteristics (i.e. attributes). It is assumed that an individual's preference for an alternative is based on the values of the attribute levels. Individuals' preferences can be inferred from the choices made across multiple choice-tasks. Using a DCE, researchers can quantify the importance of preferred treatment characteristics, calculate willingness-to-pay or willingness-to-accept risks and estimate potential participation rates, which makes this method highly attractive and superior to competing methodologies [25,26]. An example of a choice task for our study is included below in *Table 1*. The design and analysis of this survey will follow best practices [32,33]. Since a DCE can only include a total of about 5-7 of the most important attributes, it will not provide a fully holistic overview of the importance of all characteristics of gNBS on the decision to participate in such a screening procedure. However, using only the most important attributes results, uptake behaviour can be correctly predicted as the positive predictive value of DCEs has been shown to be over 0.90 (De Bekker-Grob et al., 2020)

Table 1. Example choice task DCE.

	Screening A	Screening B	No screening
Accuracy of screening test	90%	85%	Not applicable
Waiting time between test and results	2 weeks	1 month	NA

For peer review only - http://bmjopen.bmj.com/site/about/guidelines.xhtml

Health care professional informing you about results	Nurse	General Practitioner	NA
I prefer:	0	0	0

Note: This figure is for illustrative purposes only. The final choice task will be determined after considering information learned from the interview and focus groups. A "no screening" alternative will be included in the DCE study since this option is necessary to estimate uptake of screening alternatives, including the proportion who would opt out of screening.

This prospective, non-interventional, cross-sectional study will be designed in a stepwise manner [28]. First, a systematic literature review will be performed to develop an initial assessment of the perspectives and attitudes of healthcare professionals and parents regarding gNBS for RDs. Data extraction forms were designed by researchers involved in this process (11 duos of experts from the S4C project). Following the initial selection, an external firm specializing in various databases and JBI tools, LUCID ltd, located in Marlow, England, conducted a scientific quality evaluation (appraisal scoring). Subsequently, only those articles that exhibited a quality level above the average in their respective category were selected for further analysis. Data was analysed using thematic analysis. The main outcome of the review was a list of characteristics of gNBS that impact decisions of parents to participate in such screening or not (i.e., the list of potential attributes)[3].

The qualitative research step will gather data using both focus groups and individual interviews. These two different types of interviews will collect both explorative and in-depth data. Focus groups will be conducted with expecting parents seeking genetic consultation as well as "healthy" expecting parents, while individual interviews will be conducted with medical geneticists. The individual interviews and focus groups are expected to start in 2023, and to be completed before June 2024. During all interviews, a structured interview guide will be followed by trained interviewers. Before the start of the interviews, participants will be asked to sign an informed consent form (paper version if the group is held in person, or online form version if the group is held via video conference) and complete a short demographics survey (age, gender, number of previous pregnancies (expecting parents only) and years of experience (clinicians only)). The focus groups and individual interviews will follow the structure of the nominal group technique (NGT; see [26]). After several introductory questions, participants

[3] The output of the systematic literature review is planned to be published separately as a stand-alone paper in a scientific journal.

For peer review only - http://bmjopen.bmj.com/site/about/guidelines.xhtml

will be asked to rank a list of potential attributes (emanating from the literature review) from most to least important according to their personal willingness to take part in gNBS. These rankings will be discussed, and, following the discussions, participants will be allowed to change their ranking. Patient representatives from 11 organizations, who are a member of the S4C Patients Advisory Board (PAB) and joined the consortium to ensure that the perspective of PLWRDs is secured and given priority in all research activities of Screen4Care, will review the interview guides and short demographics survey to provide feedback and make sure the wording and definitions used are understandable and appropriate.

Based on the attribute ranking in the qualitative portion of the study, the DCE will be designed. This DCE will be part of a larger survey including background questions and clinical scales (see *section Data Analysis*). Given the number of attributes and levels included in the DCE, respondents would not be able to complete a full factorial design (i.e., over 1000 choice tasks). Most DCEs have respondents answer 8-15 choice questions. Blocked designs offer the opportunity to increase the efficiency of the data collection while the cognitive load on respondents stays low ass they only complete a subsection (block) of the total number of choice tasks. Therefore, such designs are typical in DCEs (Johnson et al., 2013). When blocking a design, all respondents will still see all attributes and levels but only a subset of combinations. The blocks of questions each respondent receives will be randomized to mitigate any potential bias of the blocked design while maximizing statistical power of the full design in the total study population (Hensher et al., 2015).

The DCE itself will be constructed using a Bayesian D-efficient design with choice tasks consisting of two hypothetic gNBS alternatives and a “no screening” alternative [25,34]. The educational section of the survey will be designed in close collaboration with patient organisation partners (PAB and EURORDIS representatives) similarly to the interview guides, to ensure that the information is accurate and clear to all participants, and assuring readability and appropriateness of terms. Following development of the instrument the survey will be pretested amongst expecting parents seeking genetic consultation, “healthy” expecting parents, and medical geneticists to ensure that the survey is understandable to the same population that will take the final online programmed survey in domestic languages.

Study Population and recruitment

For the qualitative part of this study three groups of interviewees are identified: (1) medical geneticists (individual interviews), (2) “healthy” expecting parents from the general population

For peer review only - http://bmjopen.bmj.com/site/about/guidelines.xhtml

(focus groups), and (3) expecting parents currently seeking genetic consultation (focus groups). These 3 groups will be recruited both in Germany and in Italy. All participants must be between the age of 18 and 70 years. Furthermore, they must be able to understand and speak German or Italian respectively as all interviews and survey material will be provided in native country-specific language (German or Italian). German and Italian procedures and methods are illustrated in Figure 1 and Figure 2.

Distribution of participant group to study phases:

Medical geneticists are considered to be experts in precise genetic screening, advanced interpretation and results delivery to HCPs teams and patients. Medical geneticists involved in genetic screening will be recruited via purposive sampling within clinical partners of Screen4Care project and be interviewed as experts. The results of these interviews will not lead to any specific analyses but ensure the adequacy of the interview guide and questions.

The second group consists of "healthy" expecting parents from the general population (with pregnancy monitoring in regular care) recruited in the same geographic areas as the expecting parents currently seeking genetic consultation for RDs in one of the clinical centres. Focus group will be run by trained personal at the recruiting centre (trained midwifes, research nurses, depending on available resources). Uppsala University will support in the implementation and conduct of the group interviews. As a precautionary measure, if both partners volunteer to the study; the mother and father from the parental couple, will be included in separate groups to avoid any influence they may have on each other during the discussions. Recruitment of "healthy" expecting parents will be conducted via midwifery and gynaecology practices in Germany and in Italy.

The third group of participants will include expecting parents who seek genetic consultation but who are not necessarily carriers of genetic variants related to RDs themselves.. Recruitment of expecting parents seeking genetic consultation will be conducted by clinical genetic centres that are part of the Screen4Care project in both Germany and Italy. Parents will be identified as they visit the clinical centres.

For the DCE survey, "healthy" expecting parents from the general population and expecting parents already seeking genetic consultation will be recruited. "Healthy" parents

For peer review only

from general population will be recruited via SurveyEngine[4] in Italy, and via invitation shared from participating centres networks in Germany. Parents already seeking genetic consultation will be recruited via invitation from the participating clinical centres in Germany and Italy. The study aims to recruit approximately 2084 participants in total across the two participating countries (see Figure 3). For the qualitative phase, a maximum sample of 4 experts (two in Germany and two in Italy), 40 expecting parents (representing 20 couples in each country) for the group interviews will be recruited. The recruitment will be operated by the clinical centres that have access to "healthy" expecting parents (total of 84 for the first part). For the quantitative phase, the survey sample will be about 1000 participants in each country (500 "healthy" expecting parents from general population and 500 expecting parents seeking genetic consultation) (see figure 1 and 2).

A priori sample-size calculations represent a challenge in DCE experiments. Most published choice experiments have a sample size of 10 to more than 3000 respondents [35]. However, minimum sample size depends on several criteria, including the question format, the complexity of the choice task, the desired precision of the results, and the need to conduct subgroup analyses [32]. A method for computing sample size was proposed by de Bekker-Grob et al., 2015 [33], however, as the article points out there is no analytic solution or power calculation that can be used to determine the appropriate sample size for a DCE unless the researcher has enough information to inform the selection of priors. Additionally, for the "healthy" expecting parents from general population sample we plan to use a blocked design (asking people to only complete a randomly assigned subset of the choice tasks, thereby limiting the cognitive burden on each participant). Given what we know, recruiting 1000 respondents in each country should be sufficient to answer the proposed research questions and provide enough information to identify preferences in these groups and comparisons across these groups with acceptable precision.

Adapting Methods to Ethical specificities

As German, and Italian regulations differ in their understanding of the risk level of focus group in terms of privacy and discomfort for the participants (e.g. genetic related conversation that could lead to sharing opinion or experience connected with genetics in front of other participants might be an argument for ethical committees' refusal in Italy). Methods have been

[4] Survey Engine is a third-party recruitment company. https://surveyengine.com/

For peer review only

adapted for example by conducting individual interviews for parents seeking genetic screening (more sensitive sample) only by clinical geneticist (in Italy). Any informational material provided before signing the informed consent will point out the specific risks and expected benefits for participants to take an informed decision before entering the protocol.

Study Procedures

Information of participants and informed consent

Each participant will receive detailed written information on the nature, objectives, possible risks/ benefits of the research (for both the focus groups and quantitative preference study participation). Prior to the qualitative interviews, participants will be given time to consider their involvement and ask questions about the study to the researchers. They will be informed that they can stop their participation in the study at each point. Participants can only take part in the study if informed consent form is signed beforehand. The investigators will keep the original signed informed consent in accordance with current requirements. For online survey participation, the informed consent will be included in the online survey and digitally signed by the participants.

Individual interviews

Semi-structured interviews will be conducted with members of the IMI S4C consortium to test the interview guide and associated questions. These interviews will be conducted in English. The interview will take approximately one hour and will be conducted at a location convenient for the interviewee, via telephone, or via video conferencing platform.

Feedback from the pilot interviews will be evaluated and the interview guides will be adapted accordingly for each country. After making the necessary changes, the interview guide will be translated to German and Italian. Native language speaking trained interviewers from clinical centers will conduct the interviews using the interview guide. Collection, recording, and reporting of data will ensure the privacy, health, and welfare of research subjects during and after the study and in accordance with the country-specific regulations.

Group interviews

For peer review only

The group interviews will be used to provide more precise evidence for the most important characteristics of gNBS when parents decide about their participation in genetic screening programs.

At the start of the interview the objective of the S4C project and the interview procedures will be explained to the interviewee and the researcher will confirm that informed consent was given before starting the interview. The interviewee must sign the consent form before the start of the interview. Once informed consent is confirmed, the researcher will start the recording device. After asking the questions in the interview guide, participants will be asked to rank a list of potential attributes (emanating from the literature review) from most to least important according to their personal decision to take part in gNBS. These rankings will be discussed, and following the discussions participants will be allowed to change their ranking. The interviewer will ask the interviewees if they want to add something to the interview.

We plan to conduct a minimum of four group interviews per country (Germany, Italy) (see Figure 1, 2 and for overall description see Figure 3) and continue until data satuarion. There will be eight group interviews, in total. A group interview consists of 8-10 members. The group interview will take about two hours and will be conducted at a location and on date most convenient for all participants.

Figure 1: Study Flowchart in Germany GOES HERE

Figure 2: Study Flowchart in Italy GOES HERE

Figure 3: Protocol's procedures GOES HERE

The interviews will be recorded and transcribed verbatim. The interviews will be analysed iteratively using a grounded theory approach based on a mix of inductive and deductive coding. Analyses will be conducted in *Atlas.ti* software. The coding procedure will be handled by two independent coders from each country. The outputs will then be translated into English for further use by the research team. To assure a proper standardisation of the interviews across languages, the domestic researchers will be trained by Uppsala's University researchers and

For peer review only

will also be provided a detailed interview guide to help them through the process of every interview.

The information given by the interviewee will be treated as confidential and will be processed anonymously. The collected data will be stored securely and viewed only by authorised researchers of the project. The processed answers will be used for publication of reports or articles as part of the S4C research project. The audio recordings specifically will be destroyed after completion of the study and no files will be stored at the recruiting centers.

Discrete Choice Experiment (DCE)

A survey will be fully developed after the final selection of attributes and levels. The final selection of attributes (and levels) to be included in the DCE will be based on the (focus group) interviews with medical geneticists and PLWRD's advocates. First, attributes ranked highest during the qualitative studies will be taken forward for consideration. For attributes to be included in the final DCE they should be unambiguous, not overlapping and clearly defined. Additionally, they should be important to the anticipated target population while at the same time clinically relevant. Therefore, the final selection of the most important attributes will be based on attribute ranking from the focus groups in combination with the perspective of experts on practicalities of gNBS. After selecting the final attributes, appropriate levels that reflect current practice will inform the attributes list for the DCE.

The survey instrument, including the DCE, will be pre-tested using think aloud methods in a convenience sample of 3 to 5 "healthy" expecting parents from the general population in each country, using think aloud interviews to review the results from the interviews and to determine if any adjustments need to be made to improve clarity. This process will be conducted in collaboration with EURORDIS and patient organisation representatives' members of the PAB. They will ensure accurate use of vocabulary by assisting in drafting and reviewing the included definitions and explanatory texts as well as educational content to ensure accessibility and readability to lay audiences.

The initial DCE experimental design will be generated via NGene 1.0 (ChoiceMetrics, 2011) and hypothetical choice questions with three alternatives will be created (of which one is the 'no screening' alternative). These alternatives will be characterised by varying attribute levels. To ensure design efficiency, a pilot with 100 respondents in each country will be

For peer review only

conducted. All participants will provide informed consent before entering the task. Based on the data retrieved in the pilot test, a multinomial logit (MNL) model will be fitted. Beta estimates will be used to assign priors for the final experimental DCE design, which is a *d*-efficient (Bayesian) design [29]. To increase efficiency of the design, multiple blocks of choice tasks will be generated which will later be randomly distributed of respondents in such a way that each respondent will answer only one block of approximately 10-15 choice tasks.

Ethical and legal aspects

The S4C IMI Project (grant agreement No. 101007757) has been approved by the Medical Ethics Committee of References. **Ethics and dissemination**: This study has been approved by the Erlangen University Ethics Committee (22-246_1-B), the Freiburg University Ethics committee (23-1005-S1-AV) and for clinical centers in Italy (University of FerraraCE: 357/2023/Oss/AOUFe, and Hospedale Bambino Gesu: No.2997 of 2 November 2023, Prot. No. _902), and approved for data storage and handling at Uppsala University (2022-05806-01). The dissemination of the results will be ensured via scientific journal publication (Open Access).

The ethical procedure will be performed at two distinct stages, the first for the qualitative part and the second for the quantitative part (DCE). As the first part will be used as an input and will inform the second one, ethical committee may not consider approving a "tentative" survey draft or a "tentative" list of attributes as it can significantly change the ethical risk for anonymity (if one attribute is "cost" compared to "pregnancy planning"). The overall S4C project also ensured an ethical framework with the Code of Ethics Practice that was developed internally as a project Deliverable and agreed with all WP leaders to inform each researcher within the S4C consortium about the main ethical risks with no exception of this specific task on preferences studies.

Confidentiality

Due to the interaction of the researchers with the interviewees it is impossible to collect the data anonymously by the recruiting centers, but the obtained information will be coded to allow confidential and anonymous processing and reporting of the data from the research team. The identity of the participants will not be revealed so the privacy will be guaranteed. The research data will be used for publications in scientific journals and writing of reports or

For peer review only

publication for the S4C project but will be registered and saved with a secure code. The coded anonymous data can be shared within the members of the consortium. The code, being the identification numbers linked to the identity of the participants, will be held confidential within the group of involved researchers. Only the researchers that are involved in this study will have access to this code and only they will know the identity of the participating stakeholder members.

Uppsala University will provide secure data storage space with restricted access to researcher analysing the data from the S4C partners involved in task 3.1. All digital data will be stored in Uppsala University's officially storage of research data (Dataportal Allvis) with specific ethical approval from The Swedish Ethical Review Authority, as Swedish Regulation requires. The data is physically stored in two redundant datacentres following (non-certified) ISO 27001 guidelines. Dataportal Allvis meets KRT value 233. Both datacentres are equipped with enterprise grade network, servers and hard disks with high availability, security and safety. All data centres are equipped with access controls, cooling systems, backup power and surge protection. They are protected against common threats such as fire, water damage, burglary, and theft. Access is restricted to technical staff and strictly protocolled. All data is encrypted in transit (https) and at rest (through database encryption). Allvis is configured with versioning of files, saving up to 500 versions of an individual file. A recycle bin saves deleted files up to 30 days. Content databases are configured with transactional logging, limiting potential data losses to 1 hour during critical failures. Databases are backed up incrementally, daily, weekly, monthly, and yearly using a TSM-based backup system hosted in the same data centers. Network traffic is protected through a robust firewall and servers hosting file content are not directly connected to the internet. The involved researchers commit to the highest standards of data security and protection to preserve the personal rights and interests of study participants. Participants will be informed that they can contact Uppsala University data security services for any information via email (dataskyddsombud@uu.se).

The involved researchers commit to the highest standards of data security and protection to preserve the personal rights and interests of study participants. They will adhere to the provisions set out in:

- The General Conference of the United Nations Educational, Scientific and Cultural Organization (UNESCO), meeting in Paris, from 9 to 24 November 2021, at its 41st session for Open science recommendations.

For peer review only - http://bmjopen.bmj.com/site/about/guidelines.xhtml

- A central guide for all research that includes human subjects will be the Declaration of Helsinki - Ethical principles for medical research involving human subjects from the World Medical Association (WMA Adopted by the 18th WMA General Assembly, Helsinki, Finland, June 1964 -amended by the 64th WMA General Assembly, Fortaleza, Brazil, October 2013
- Directive 2002/58/EC of the European Parliament and of the Council of 12 July 2002 concerning the processing of personal data and the protection of privacy in the electronic communications sector (Directive on privacy and electronic communications)
- Regulation (EU) 2016/679 of the European Parliament and of the Council of 27 April 2016 on the protection of natural persons regarding the processing of personal data and on the free movement of such data, and repealing Directive 95/46/EC (General Data Protection Regulation) (Text with EEA relevance)
- Regulation (EU) 2018/1725 of the European Parliament and of the Council of 23 October 2018 on the protection of natural persons regarding the processing of personal data by the Union institutions, bodies, offices, and agencies and on the free movement of such data, and repealing Regulation (EC) No 45/2001 and Decision No 1247/2002/EC

Data analysis

Using the systematic literature review, a list of tentative attributes will be extracted from the thematic analysis based on gNBS themes and recurrent factors. The list will also include psychological factors (e.g. depression, anxiety, emotion regulation) that will have to be addressed as contextual elements separately. As the literature extensively points at the psychosocial impact of gNBS, even in 2023 (Blom et al., 2023; Tobik et al., 2023), we decided to include the main psychological measures to ensure possibility for subgroup analysis based on validated measures to better describe the sample but also be able to potentially describe differences in preferences within and between the populations.

As numerous potential attributes are expected to be highlighted during the literature review, the focus group will help clarify the most important elements. Independent coders will analyse the transcription of the focus group and provide content analysis. In order to refine the attribute list, the ranking task will be performed presenting 10-15 attributes to design the DCE. The data from the ranking task will be analyzed to understand what attributes are most

For peer review only

important to expecting parents. This information will be considered when deciding the final attributes for the DCE.

Participant characteristics that will be reported to describe the study samples include demographic characteristics (age, gender, pregnancy stage, income level among others), but also attitudes and psychosocial factors such as health literacy, and anxiety for example (Hospital Anxiety and Depression Scale, available in both Italian and German; Annunziata et al., 2011; Hinz & Brähler, 2011). In collaboration with recruiting centers, we will consider the inclusion of personality scales, emotion regulation scales and-or coping strategies scales to provide a more precise psychological assessment.

Preferences and understanding of the survey instrument (comprehension questions) will be assessed by examining the relationship between psychological instruments of cognitive ability (e.g. health literacy and numeracy) and survey responses. This includes comparing responses to warm up questions, survey internal validity checks, and attitudinal questions about reported complexity of tasks across various levels of each psychological instrument.

To determine preferences of study participants regarding gNBS, attributes, level estimates and the conditional relative importance of attributes will be reported in the DCE using random parameters logit (RPL) modelling and latent class analyses (LCA). Heterogeneity of preferences and the impact of participant characteristics (e.g. demographics, psychological validated scales) will be investigated by applying appropriate statistical models including LCA for the DCE and/or subgroup analyses (RPL). All results described above will be compared between the German and Italian study samples to determine whether there are cross-country differences. The results of the quantitative part of the preference study is planned to be published in scientific journal (Open Access) and to be shared in all S4C project communication networks to reach out to different audiences as part of the general communication plan of the project.

For peer review only

Acknowledgements:

Author contributions: Sylvia Martin (SM), Joshua Coulter (JC), Jorien Veldwijk (JV) drafted the protocol and closely ensures the adaptation of the final version to all authors's comments.

SM, JC, JV, Åsa Grauman (AG), Brett Hauber (BH), Mats Hansson (MH), contributed to the design and implementation of the protocol.

Planning of the work, conduct and reporting was ensure by all authors and included clinicians for the process of ethical application and procedure, but also to design the protocol in adequacy with clinical requirements. Ethical procedure was led by SM, MA, Angolini Emanuele (AE), Enrico Silvio Bertini (ESB), Lucia Pia Bruno (JPB), Alessandra Ferlini (AF), Fernanda Fortunato (FF), Vera Frankova (VF), Janbernd Kirschner (JK), Ferdinand Knieling (FK), Silvia Ottombrino (SO), Antonio Novelli (AN), Roman Raming (RR), Christina Saier (CS), Stefaan Sansen (SS) and Nicolas Garnier (NG). Edith Gross (EG) coordinated the patients and patient's advocacy participation for both ethical procedure and protocol drafting.

SM, Gergana Kyosovksa (GK), Jennifer Audi (JA), drafted the first version of the project and included all relevant ethical consideration and introduction information.

FK, RR, JK, SS, CS, LPB, VF, EG, JA, GK, FF, AF, NG, ESB, AE, SO, AN, SM, JC, JV, AG, BH, MH, drafted the precise clinical information used in the draft, checked for relevance of the vocabulary and reviewed the overall structure of the article.

Non-authors contribution: We thank the members of the Screen 4 Care Patient advisory board for their constructive help during consultation meeting and for the quality of their written feedback.

Funding statement: 'This work was supported by Sreen4Care. This project has received funding from the Innovative Medicines Initiative 2 Joint Undertaking (JU) under grant agreement **No 101034427**. The JU receives support from the European Union's Horizon 2020 research and innovation programme and EFPIA.

Competing interests' statement: No competing interest.

Data statement: Data will be made available under reasonable demand to the corresponding authors and in accordance to Screen 4 Care data security procedures.

For peer review only - http://bmjopen.bmj.com/site/about/guidelines.xhtml

References:

[1] Nguengang Wakap, S., Lambert, D. M., Olry, A., Rodwell, C., Gueydan, C., Lanneau, V., Murphy, D., Le Cam, Y., & Rath, A. (2020). Estimating cumulative point prevalence of rare diseases: analysis of the Orphanet database. *European journal of human genetics: EJHG*, *28*(2), 165–173. https://doi.org/10.1038/s41431-019-0508-0

[2] Hay E, Elmslie F, Lanyon P and Cole T. The Diagnostic Odyssey in rare diseases; a Task and Finish Group report for the Department of Health and Social Care [version 1; not peer reviewed]. *NIHR Open Res* 2022, **2**:3 (document) (https://doi.org/10.3310/nihropenres.1115171.1)

[3] Marwaha, S., Knowles, J. W., & Ashley, E. A. (2022). A guide for the diagnosis of rare and undiagnosed disease: beyond the exome. *Genome medicine*, *14*(1), 1-22.

[4]Ferreira C. R. (2019). The burden of rare diseases. *American journal of medical genetics. Part A*, *179*(6), 885–892. https://doi.org/10.1002/ajmg.a.61124

[5] Gokdemir, Y., Eyuboglu, T. S., Emiralioglu, N., Er, B., Sen, V., Pekcan, S., ... & Yılmaz, O. (2021). Geographical barriers to timely diagnosis of cystic fibrosis and anxiety level of parents during newborn screening in Turkey. *Pediatric Pulmonology*, *56*(10), 3223-3231.

[6] Grob, R., Roberts, S., & Timmermans, S. (2018). Families' Experiences with Newborn Screening: A Critical Source of Evidence. *Hastings Center Report*, *48*, S29-S31.

[7] Bailey Jr, D. B., Bishop, E., Raspa, M., & Skinner, D. (2012). Caregiver opinions about fragile X population screening. *Genetics in Medicine*, *14*(1), 115-121.

[8] van Dijk, T., Kater, A., Jansen, M., Dondorp, W. J., Blom, M., Kemp, S., ... & Henneman, L. (2021). Expanding Neonatal Bloodspot Screening: A Multi-Stakeholder Perspective. *Frontiers in pediatrics*, *9*, 706394.

[9] Chavez-Yenter, D., Vagher, J., Clayton, M. F., Rindler, M., Shukovich, M., & Kaphingst, K. A. (2021). "Being proactive, not reactive": exploring perceptions of genetic testing among White, Latinx, and Pacific Islander Populations. *Journal of Community Genetics*, *12*(4), 617-630.

[10] Perobelli, S., Zanolla, L., Tamanini, A., Rizzotti, P., Maurice Assael, B., & Castellani, C. (2009). Inconclusive Cystic Fibrosis neonatal screening results: long-term psychosocial effects on parents. *Acta Paediatrica*, *98*(12), 1927-1934.

[11] Pruniski, B., Lisi, E., & Ali, N. (2018). Newborn screening for Pompe disease: Impact on families. *Journal of inherited metabolic disease*, *41*(6), 1189-1203.

[12] Bailey Jr, D. B., Berry-Kravis, E., Gane, L. W., Guarda, S., Hagerman, R., Powell, C. M., ... & Wheeler, A. (2017). Fragile X newborn screening: lessons learned from a multisite screening study. *Pediatrics*, *139*(Supplement_3), S216-S225.

[13] Friedman, J.M., Cornel, M.C., Goldenberg, A.J. *et al.* Genomic newborn screening: public health policy considerations and recommendations. *BMC Med Genomics* **10**, 9 (2017). https://doi.org/10.1186/s12920-017-0247-4

[14] Knoppers, B. M., Sénécal, K., Borry, P., & Avard, D. (2014). Whole-genome sequencing in newborn screening programs. *Science translational medicine*, *6*(229), 229cm2-229cm2. DOI: 10.1126/scitranslmed.3008494

For peer review only - http://bmjopen.bmj.com/site/about/guidelines.xhtml

[15] Ozdemir, S., Finkelstein, E., Lee, J. J., Too, I. H. K., Teo, K. Y. C., Tan, A. C. S., ... & Cheung, G. C. M. (2022). Understanding patient preferences in anti-VEGF treatment options for age-related macular degeneration. *PloS one*, *17*(8), e0272301.

[16] Moultrie, R. R., Paquin, R., Rini, C., Roche, M. I., Berg, J. S., Powell, C. M., & Lewis, M. A. (2020). Parental views on newborn next generation sequencing: implications for decision support. *Maternal and child health journal*, *24*, 856-864.

[17] Bombard, Y., Miller, F. A., Hayeems, R. Z., Barg, C., Cressman, C., Carroll, J. C., ... & Chakraborty, P. (2014). Public views on participating in newborn screening using genome sequencing. *European Journal of Human Genetics*, *22*(11), 1248-1254.

[18] Plass, A. M. C., van El, C. G., Pieters, T., & Cornel, M. C. (2010). Neonatal screening for treatable and untreatable disorders: prospective parents' opinions. *Pediatrics*, *125*(1), e99-e106.

[19] Bailey, D. B., Lewis, M. A., Harris, S. L., Grant, T., Bann, C., Bishop, E., ... & Therrell, B. L. (2013). Design and evaluation of a decision aid for inviting parents to participate in a fragile X newborn screening pilot study. *Journal of genetic counseling*, *22*(1), 108-117.

[20] Miller, F. A., Hayeems, R. Z., Bombard, Y., Cressman, C., Barg, C. J., Carroll, J. C., ... & Regier, D. A. (2015). Public perceptions of the benefits and risks of newborn screening. Pediatrics, 136(2), e413-e423.

[21] Wright, S. J., Ulph, F., Lavender, T., Dharni, N., & Payne, K. (2018). Understanding midwives' preferences for providing information about newborn bloodspot screening. *MDM policy & practice*, *3*(1), 2381468317746170.

[22] Loeber, J. G., Platis, D., Zetterström, R. H., Almashanu, S., Boemer, F., Bonham, J. R., Borde, P., et al. (2021). Neonatal Screening in Europe Revisited: An ISNS Perspective on the Current State and Developments Since 2010. *International Journal of Neonatal Screening*, *7*(1), 15. MDPI AG. Retrieved from http://dx.doi.org/10.3390/ijns7010015

[23] Hammond, J., Klapwijk, J. E., Riedijk, S., Lou, S., Ormond, K. E., Vogel, I., ... & Lewis, C. (2022). Assessing women's preferences towards tests that may reveal uncertain results from prenatal genomic testing: Development of attributes for a discrete choice experiment, using a mixed-methods design. *PloS one*, *17*(1), e0261898.

[24] Buchanan, J., Hill, M., Vass, C. M., Hammond, J., Riedijk, S., Klapwijk, J. E., ... & Lewis, C. (2022). Factors that impact on women's decision-making around prenatal genomic tests: An international discrete choice survey. *Prenatal diagnosis*, *42*(7), 934-946.

[25] Hensher, D., J.M. Rose, and W.H. Greene, *Applied Choice Analysis: second edition*. 2015, Cambridge, UK: Cambridge University Press.'

[26] Hiligsmann, M., et al., Nominal group technique to select attributes for discrete choice experiments: an example for drug treatment choice in osteoporosis. Patient Prefer Adherence, 2013; 7:133-9

[27] Reed Johnson, F., E. Lancsar, D. Marshall, V. Kilambi, A. Muhlbacher, D.A. Regier, B.W. Bresnahan, B. Kanninen, and J.F. Bridges, Constructing experimental designs for discrete-choice experiments: report of the ISPOR Conjoint Analysis Experimental Design Good Research Practices Task Force. *Value Health*, 2013. 16(1): p. 3-13.

[28] Bridges, J.F., A.B. Hauber, D. Marshall, A. Lloyd, L.A. Prosser, D.A. Regier, F.R. Johnson, and J. Mauskopf, Conjoint analysis applications in health--a checklist: a report of the

For peer review only - http://bmjopen.bmj.com/site/about/guidelines.xhtml

ISPOR Good Research Practices for Conjoint Analysis Task Force. *Value Health*, 2011. 14(4): p. 403-13.

[29] Kessels, R., et al., An efficient algorithm for constructing bayesian optimal choice designs. Journal of Business & Economic Statistics, 2009; 27(2):279-291.

[30] Louviere, J.J., et al., Designing discrete choice experiments: do optimal designs come at a price? Journal of Consumer Research, 2008; 35(2):360-375.

[31] Marshall, D., et al., Conjoint Analysis Applications in Health - How are Studies being Designed and Reported?: An Update on Current Practice in the Published Literature between 2005 and 2008. Patient., 2010; 3(4):249-56.

[32] Louviere, J., D. Hensher, and J. Swait, Stated Choice Methods; analysis and application. 2000, Cambridge: Cambridge University Press.

[33]de Bekker-Grob, E.W., B. Donkers, M.F. Jonker, and E.A. Stolk, Sample Size Requirements for Discrete-Choice Experiments in Healthcare: a Practical Guide. *Patient*, 2015. 8(5): p. 373-84.

[34] Rose, J. M., & Bliemer, M. J. C. (2009). Constructing efficient stated choice experimental desings. *Transport Reviews, 9, 1–31*.

[35] Merlo, G., van Driel, M., & Hall, L. (2020). Systematic review and validity assessment of methods used in discrete choice experiments of primary healthcare professionals. *Health economics review*, *10*(1), 1-9.

For peer review only - http://bmjopen.bmj.com/site/about/guidelines.xhtml

Figure Legend:

Figure 1- Study Flowchart in Germany

Figure 2- Study Flowchart in Italy

Figure 3- Protocol's procedures

For peer review only

For peer review only - http://bmjopen.bmj.com/site/about/guidelines.xhtml

For peer review only

Figure 1: Study Flowchart in Germany

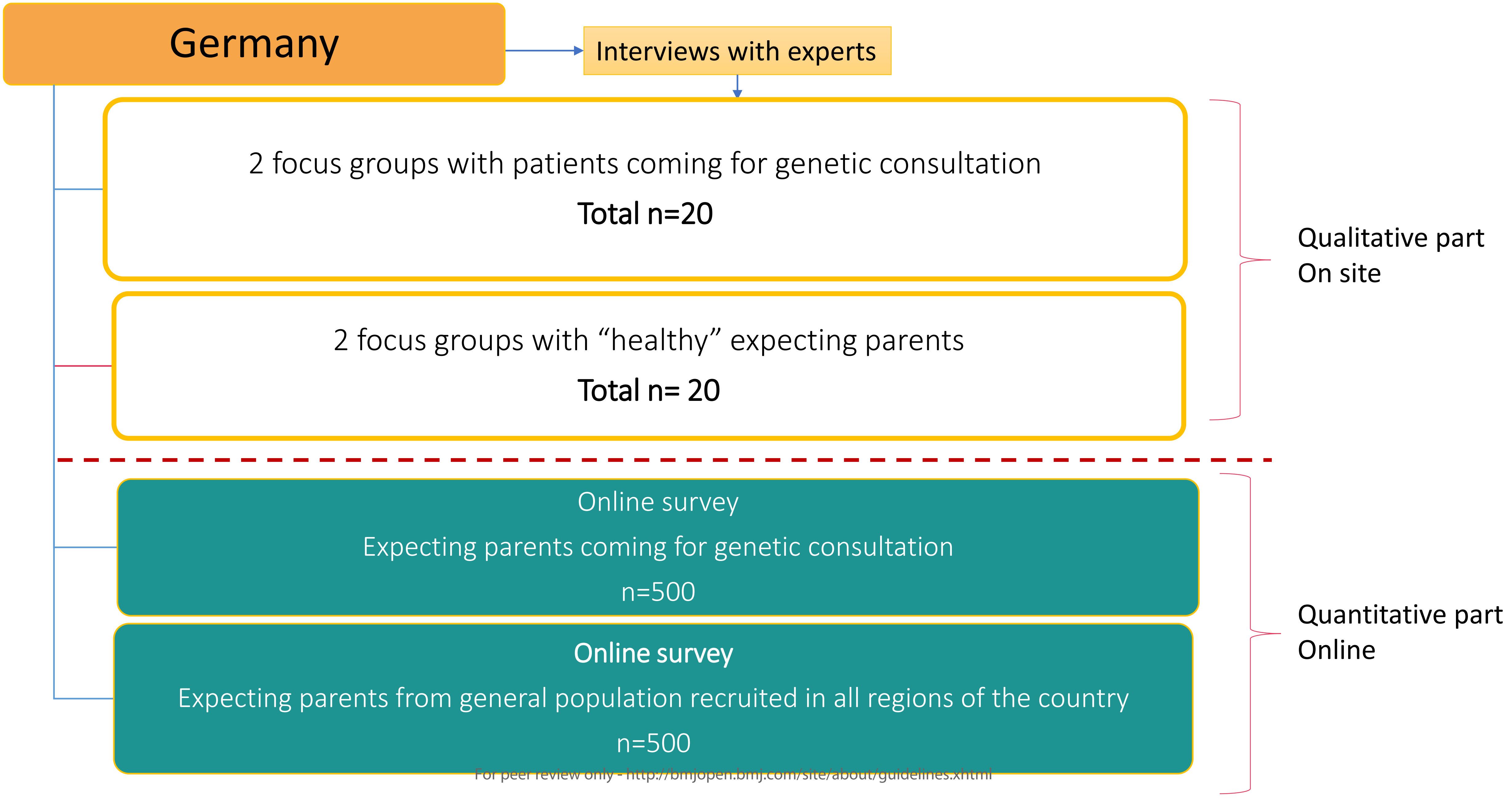

Figure 2: Study Flowchart in Italy

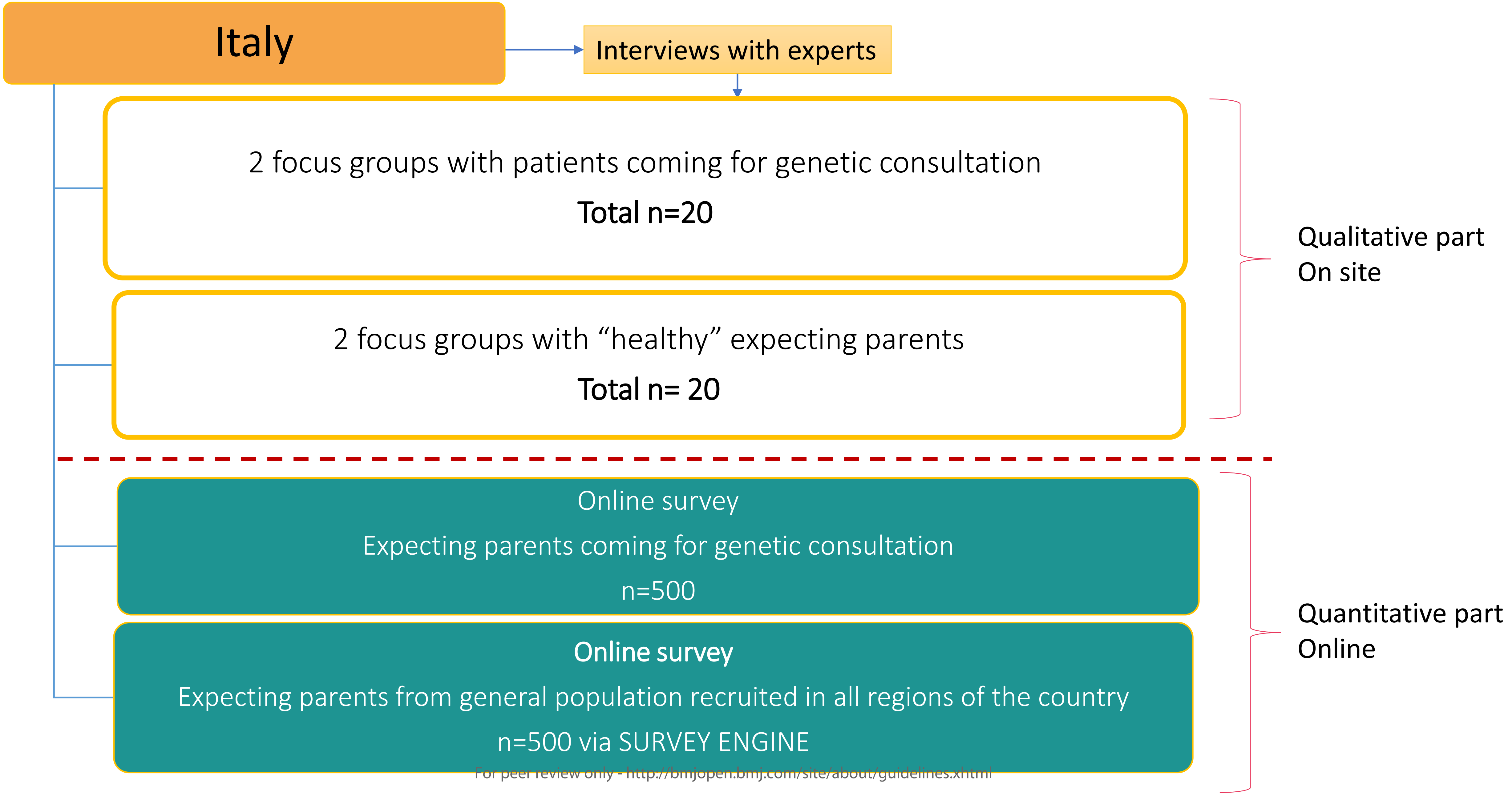

Figure 3: Protocol's procedures

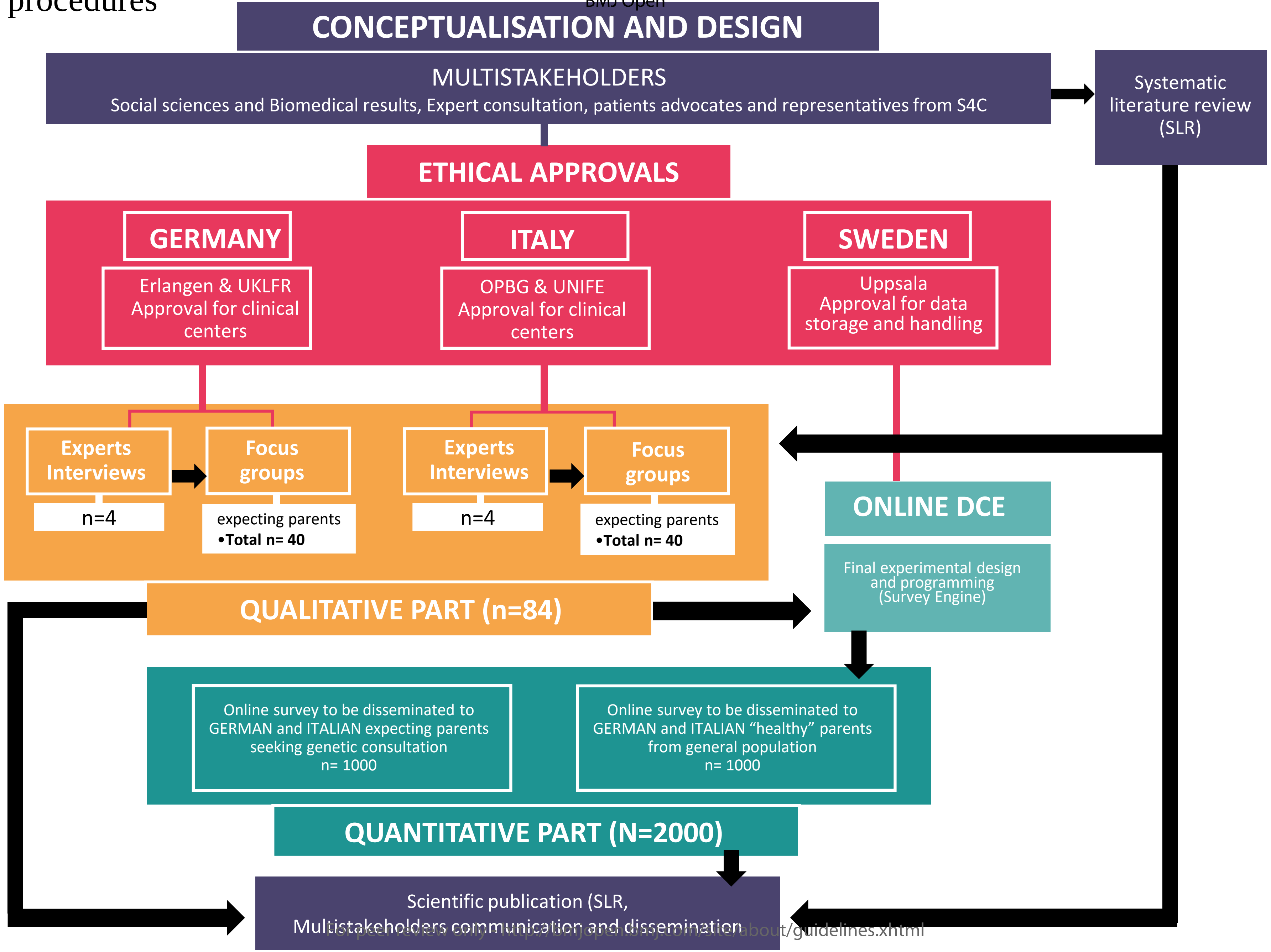

For peer review only - http://bmjopen.bmj.com/site/about/guidelines.xhtml
